# The virulence domain of *Shigella* IcsA contains a subregion with specific host cell adhesion function

**DOI:** 10.1371/journal.pone.0227425

**Published:** 2020-01-07

**Authors:** Jilong Qin, Matthew Thomas Doyle, Elizabeth Ngoc Hoa Tran, Renato Morona

**Affiliations:** School of Biological Sciences, Department of Molecular & Biomedical Sciences, Research Centre for Infectious Diseases, University of Adelaide, Adelaide, Australia; New York State Department of Health, UNITED STATES

## Abstract

*Shigella* species cause bacillary dysentery, especially among young individuals. Shigellae target the human colon for invasion; however, the initial adhesion mechanism is poorly understood. The *Shigella* surface protein IcsA, in addition to its role in actin-based motility, acts as a host cell adhesin through unknown mechanism(s). Here we confirmed the role of IcsA in cell adhesion and defined the region required for IcsA adhesin activity. Purified IcsA passenger domain was able block *S*. *flexneri* adherence and was also used as a molecular probe that recognised multiple components from host cells. The region within IcsA’s functional passenger domain (aa 138–148) was identified by mutagenesis. Upon the deletion of this region, the purified IcsA^Δ138–148^ was found to no longer block *S*. *flexneri* adherence and had reduced ability to interact with host molecules. Furthermore, *S*. *flexneri* expressing IcsA^Δ138–148^ was found to be significantly defective in both cell adherence and invasion. Taken together, our data identify an adherence region within the IcsA functional domain and provides useful information for designing therapeutics for *Shigella* infection.

## Introduction

Shigellae are Gram-negative bacteria that cause severe bloody diarrhoea in humans [1] and rhesus monkeys [2]. Shigellosis is life threating to children under 4 years of age [3] and is a growing health problem in developed countries due to decreased susceptibility to antibiotics [4]. *Shigella* spp. are primate specific pathogens that target the colon [1, 5] and it has been demonstrated in the rabbit ligated ileum model [6], and *in vitro* colonoids [7], that *Shigella* can be taken up by M cells. However, *Shigella* has been reported to target the human colonic crypts where M cells were not present [8], strongly indicating that an alternative route of entry exists. Indeed, the mechanisms by which *Shigella* species initially adhere to host cells, a prerequisite for subsequent invasion and establishing infection, remains poorly understood.

A role in host cell adhesion has been recently revealed for the essential surface displayed virulence factor IcsA [9]. IcsA is 100% conserved in *Shigella* species and is a member of the secreted autotransporter (AT) superfamily. IcsA contains a signal sequence (aa 1–52) at its N-terminus for secretion across the inner membrane; a passenger domain (aa 53–740) which confers its function; a β-barrel domain (aa 813–1102) which is responsible for passenger domain translocation across the outer membrane; and an unstructured linker region (aa 741–812) that connects the passenger with the β-barrel. IcsA also belongs to the AIDA subfamily with members that are well characterised adhesins, such as AIDA-I and Ag43, both of which are known to have β-helix passenger structures [10–12]. The IcsA passenger also possesses these β-helix structures [13–16]. IcsA has been well studied with respect to its function in actin based motility (ABM) [17, 18], where polarly distributed IcsA recruits host N-WASP protein [19–21], resulting in the subsequent polymerisation of host cell actin at one pole of the bacterium to facilitate bacterial inter- and intracellular motility [22]. Recently, IcsA has been found contributing to *S*. *flexneri* biofilm formation in the presence of bile salt deoxycholate (DOC) by promoting cell-to-cell contact and aggregative bacterial growth [23]. Besides ABM and biofilm formation, however, knockouts of the type 3 secretion tip complex proteins IpaD or IpaB in *Shigella flexneri* result in polar adhesion to host cells in an IcsA-dependent manner [9]. IcsA was also found to contribute to DOC induced hyper-adherence, and expressing IcsA in *E*. *coli* promotes adherence to host cells, confirming that IcsA is sufficient to promote bacterial adherence [9]. While IcsA’s expression level and cellular distribution is not altered in the hyper-adherent *Shigella* compared to the wild type strain, data suggest that the conformation of IcsA is different in hyper-adherent strains [9].

In this study, the direct role of IcsA in Shigella-host-cell adherence was demonstrated via adherence inhibition with purified IcsA passenger domain, and adherence blocking with anti-IcsA antibodies. Fluorescently labelled IcsA passenger was also able to bind to host cell surfaces. Indirect probing with purified IcsA passenger protein recognised several host molecules through far Western blotting. Through screening of an IcsA 5 aa insertion library [24], the region responsible for adhesin activity was identified and characterised.

## Materials and methods

### Ethics statement

The anti-GST antibody and anti-IcsA antibody were produced under the National Health and Medical Research Council (NHMRC) Australian Code of Practice for the Care and Use of Animals for Scientific Purposes, and was approved by the University of Adelaide Animal Ethics Committee.

### Bacterial strains and tissue culture

The bacterial strains used in this study are listed in S1 Table. For adherence assays, bacterial strains were streaked onto Tryptic Soy Agar with 0.2% (w/v) Congo Red, and after incubation at 37°C overnight, red colonies were selected and incubated in Lysogeny broth (LB) overnight with appropriate antibiotics (tetracycline, 10 μg ml^−1^; kanamycin, 50 μg ml^−1^; chloramphenicol, 25 μg ml^−1^ and ampicillin, 100 μg ml^−1^). For all assays, overnight bacterial cultures were subcultured (1:20) in the presence or absence of 2.5 mM sodium deoxycholate and grown to a mid-exponential phase (OD_600_ reading of 0.6–0.8) before use.

HeLa cells were maintained and grown in minimal essential medium (MEM) supplemented with L-glutamine, 10% (v/v) fetal calf serum (FCS), and penicillin/streptomycin. Cell cultures were maintained at 37°C with 5% CO_2_ for growth. The day prior to the bacterial adherence assay and invasion assay, or for microscopy, HeLa cells were seeded at 4.5×10^5^/well into 24-well plates or onto glass coverslips respectively. For plaque assays, HeLa cells were seeded into 6-well plates and were allowed to grow confluent.

### Mutagenesis and DNA manipulation

*S*. *flexneri* 2a *ΔipaD* or *ΔipaB* strains were generated using the λ red mutagenesis method as described previously [25]. Briefly, primers (S2 Table) were designed to PCR amplify the kanamycin cassette flanked with 50 bp of the start and the end of the coding sequences of IpaD or IpaB. The fragments were then electroporated into WT *S*. *flexneri* 2457T or a *ΔicsA* knockout strain to generate *ΔipaD/ΔipaB* or *ΔipaDΔicsA/ΔipaBΔicsA* mutant strains. The kanamycin cassette was then eliminated by the introduction of pCP20 to avoid potential polar effects.

Site-directed mutagenesis was performed on pIcsA plasmid [24] using the QuikChange II® system (Agilent) as per the manufacturers protocol. The primers used are listed in S2 Table.

For alanine scanning of the amino region 138 to 148 in the IcsA passenger domain, codons of each amino acid were substituted with a codon of alanine and changed via inverse PCR with the primers listed in S2 Table.

The hyper-adherent mutant library was generated by transforming plasmids from the IcsA 5 aa insertion library [24] into *S*. *flexneri* 2a *ΔipaDΔicsA* via chemical transformation as described by Sambrook and Russell [26].

For IcsA production, the IcsA passenger sequence from amino acid 53 to 740 was amplified using primers MD80/81 (S2 Table) from pIcsA, and cloned into the pBADhisB vector (Invitrogen) between the XhoI and KpnI sites, resulting in pBADhisB::IcsA^53-740^. The vector was then optimised for purification by inverse PCR to replace the His×6 tag with a N-terminus fused His×12 tag, resulting in pMDBAD::IcsA^53-740^. For the IcsA^Δ138–148^ production, the coding sequence of amino acids 138 to 148 in the IcsA passenger domain was deleted via inverse PCR, resulting in pMDBAD::IcsA^53-740(Δ138–148)^.

### Protein purification and refolding

For IcsA passenger domain production, an overnight culture of *E*. *coli* TOP10 transformed with pMDBAD::IcsA^53-740^ was sub-cultured 1 in 1000 into auto-induction 2 L Terrific Broth medium [27] that contained a mixture of glucose and arabinose in the ratio of 0.1%:0.3% (w/v), and incubated at 37°C overnight. Cells were then harvested by centrifugation (10,000 ×g, 10 min), resuspended in 80 ml TBS [50 mM Tris, 150 mM NaCl, pH 7.0] and lysed using a cell disruptor (30 kpsi, Constant Systems Ltd) in the presence of two EDTA-free protease inhibitor tablets (Roche). Inclusion bodies (IBs) were recovered by centrifugation of the cell lysates (20,000 ×g, 10 min), and pre-cleaned by detergent wash [50 mM Tris, 1 M NaCl, 2% (v/v) Triton X-100, 4 mM DDM and 2% (w/v) DOC, pH 8.0] to exclude membrane fractions. IcsA passenger protein from the IBs was then solubilised in 50 ml protein solubilisation buffer [8 M urea, 50 mM NaCl, 50 mM Tris, 10 mM imidazole, pH 8] for 2 h, followed by centrifugation (185,000 ×g, 1 hr). Solubilised IcsA passenger protein was then loaded on a His-trap column, washed and eluted with increasing concentration of imidazole. IcsA passenger protein was then further purified through an HiLoad 16/600 Superdex 200pg column (GE Healthcare) and eluted fractions containing purified protein were confirmed by SDS-PAGE. IcsA passenger domain-containing fractions were then pooled and subjected to refolding.

Purified IcsA passenger protein was diluted 1:20 into base buffer [50 mM NaCl, 50 mM Tris, pH 8.0] with different screening ingredients or conditions including 10% (v/v) glycerol, 1.5 M NaCl, 1% (v/v) NP-40, 0.5 M urea, 10 mM DTT, 1% (w/v) glycine, 100 mM MgCl_2_ or pH 7.0. The mixtures were then incubated for 16 h at 4°C, and ultracentrifuged (185,000 ×g, 30 min) to separate the insoluble and soluble fractions. Samples of both insoluble and soluble fractions were compared by electrophoresis into a 12% polyacrylamide SDS-PAGE gel and subjected to Western transfer and Ponceau S staining. Conditions that yielded the least aggregation were then used, and the purified IcsA passenger protein was refolded by dialysing against optimised buffer [0.5 M urea, 10% (v/v) glycerol, 50 mM NaCl and 50 mM Tris-HCl pH 7.0] at room temperature for 48 h. Dialysed IcsA passenger protein was then ultracentrifuged (185,000 ×g, 1 h) and the resulting supernatant was quantified using the protein BCA assay (Thermo Fisher) and stored at -80°C. With this protocol a yield of IcsA of approximately 10 mg protein was obtained from a 2 L overnight culture.

### Proteinase accessibility assay

Proteinase accessibility assay was performed as described by May and Morona [24] with modifications. Refolded IcsA passenger protein was incubated with Human Neutrophil Elastase (hNE, EPC Elastin Products) in dialysis buffer at the molecular ratio of 1000:1 at 37°C for 1.5 h. Aliquots were taken at different time points (0 min, 5 min, 10 min, 15 min, 30 min, 45 min, 60 min and 90 min) and immediately resuspended with an equal volume of SDS-PAGE sample buffer [29] followed by incubation at 100°C for 10 min. A sample of IcsA passenger protein was also heated at 65°C for 15 min, cooled to room temperature, and digested as above to serve as a control. Fractions taken from different time points were then analysed by SDS-PAGE and stained with Coomassie blue G250 (Sigma).

### Fluorescent labelling

For protein labelling, refolded IcsA^53-740^ protein (that has three cysteine residues available for labelling with Dylight 594 (DL^594^) maleimide), mutant IcsA protein (IcsA^53-740(Δ138–148)^), or bovine serum albumin (BSA) protein (Sigma) were incubated with DL^594^ maleimide (Thermo Fisher) at the molecular ratio of 1:2 overnight at room temperature, and subsequently dialysed against dialysis buffer to remove excessive dye. Successfully labelled protein was analysed via SDS-PAGE and the fluorescence was confirmed using a ChemiDoc imaging system (BioRad).

IcsA immunofluorescent labelling on bacterial surfaces was performed as described previously [30]. Briefly, *Shigella* grown to an OD_600_ of 0.5 was collected and fixed in PBS containing 3.7% (v/v) formaldehyde and centrifuged onto poly-L-lysine-coated coverslips. Bacteria was then incubated with rabbit anti-IcsA antibody for 1 h, washed with PBS, and labelled with Alexa 488-conjugated donkey anti-rabbit antibody for another 1 h. Samples were then mounted with 20% (v/v) Mowiol 4–88 (Calbiochem), 4 mg ml^−1^ *p*-phenylenediamine, and imaged with an Olympus fluorescent microscope (IX-70).

### SDS-PAGE and Western blotting

For SDS-PAGE, samples were resuspended in an equal volume of 2× SDS-PAGE sample buffer [29], and immediately heated at 100°C for 10 min. A total of 20 μl from each sample was then electrophoresed on Any kD^TM^ gels (BioRad) or hand-cast 12% SDS acrylamide (BioRad) gels. For Western immunoblotting, samples were then transferred onto a nitrocellulose membrane, blocked with TBST [TBS, 0.05% (v/v) Tween-20] containing 5% (w/v) skim milk, and incubated with rabbit anti-IcsA antibody [28], rabbit anti-GST antibody (in house made), or mouse anti-His antibody (Genscript) for 4 h. The membrane was then washed with TBST and incubated with HRP-conjugated goat anti-mouse antibody (Biomediq DPC) or HRP-conjugated goat anti-rabbit antibody (Biomediq DPC) for 1 h. The membrane was then washed with TBS and incubated with Chemiluminescence Substrate (Sigma) for 5 min. Chemiluminescence was detected using a ChemiDoc imaging system (BioRad).

### Confocal microscopy

To visualise the binding of IcsA to host cell surfaces, fluorescently labelled IcsA^53-740^ protein (2.8 μM), IcsA^53-740(Δ138–148)^ protein (2.8 μM) or BSA protein (5 μM) were added onto confluent HeLa cell monolayers grown on coverslips in 24-well trays and incubated at 37°C with 5% CO_2_ for 15 min followed by washing with PBS. Monolayers were then fixed with 3.7% (v/v) formaldehyde in PBS, washed with PBS, incubated with 1% (v/v) Triton in PBS for 10 min, and then stained with AlexaFluor 488 phalloidin (Invitrogen) diluted to 1:100 in PBS containing 10% (v/v) FCS. Monolayers were then washed with PBS and DNA was stained using 10 μg ml^−1^ DAPI for 1 min, followed by another PBS wash. Coverslips were then mounted with 20% (v/v) Mowiol 4–88 (Calbiochem), 4 mg ml^−1^ *p*-phenylenediamine, and imaged with an Olympus confocal laser scanning microscope (FV3000).

### Adherence, invasion and plaque formation assays

For whole cell adherence assays, *Shigella* grown to an OD_600_ of 0.4–0.6 were collected, washed with MEM, and inoculated to HeLa cell monolayers at the multiplicity of infection (MOI) of 100. Centrifugation (500 ×g, 5 min) was used as outlined in the results. After 15 min of incubation, HeLa cell monolayers were washed with PBS and lysed using PBS containing 0.1% (v/v) Triton X-100 at 37°C for 10 min. The remaining *Shigella* bacteria were enumerated by serial dilution plating onto LB agar.

For invasion assays, *Shigella* grown to an OD_600_ of 0.4–0.6 in the presence of DOC were used to infect HeLa cell monolayer at the MOI of 100. Gentamycin (40 μg ml^-1^) was added after 45 min post infection and HeLa monolayers were incubated for another 45 min before being lysed and treated as above.

Plaque formation was performed as described previously [30]. Briefly, HeLa cells grown to confluency in six-well trays were washed with PBS and Dulbecco’s modified Eagle’s medium (DMEM) sequentially before infection with of *Shigella* (1.25 ×10^5^ cfu) grown to an OD_600_ of 0.5. At 90 min post infection, an overlay [DMEM, 5% (v/v) FCS, 20 μg ml^-1^ gentamycin, 0.5% (w/v) agarose] was added to each well. The second overlay containing 0.1% (w/v) Neutral Red was added at 48 h post infection and images of plaques were taken after another 2 h incubation.

### Adherence blocking assays using purified IcsA or anti-IcsA antibody

For both adherence blocking assays, HeLa cells grown to confluence were washed with PBS, and replenished with culture medium devoid of antibiotics. For the IcsA adherence blocking assays, IcsA^53-740^ protein at different concentrations (2.5 μM, 1.25 μM, 250 nM and 25 nM) along with *S*. *flexneri* 2a strains were added onto monolayers at the MOI of 100:1. After an incubation of 15 min at 37°C with 5% CO_2_, samples were centrifuged (500 ×g, 5 min) and incubated for another 15 min as above.

For the antibody adherence blocking assays, bacteria were washed with PBS and replenished in the culture medium (as above) and incubated with either rabbit anti-IcsA pAbs (3.125 μg/ml and 0.3125 μg/ml) or rabbit pre-immune serum (concentration of IgG 100 μg/ml) for 15 min. Bacteria with antibodies were then added onto cell monolayers at the MOI of 100 and incubated for another 15 min at 37°C with 5% CO_2_. For both assays, unbound *Shigella* bacteria were washed three times with PBS, and monolayers were lysed using 0.1% (v/v) Triton X-100 at 37°C for 10 min. The remaining *Shigella* bacteria were enumerated by serial dilution plating onto LB agar.

### Statistical analysis

The statistical analysis on *Shigella* adherence and invasion assays was performed using GraphPad Prism 8.0.0. Data were normalised against the relevant control and significance was calculated using either a student *t* test or one-way ANOVA followed by Dunnett’s multiple comparisons test against the control.

### Protein lysates, cell fractionation and far Western blotting

HeLa cells grown to confluence on 100 mm dishes (approximately 8.8×10^8^ cells) were recovered either by using a cell scraper or trypsin digestion, and washed with PBS followed by centrifugation (4,000 ×g, 5 min, 4°C). Pellets were lysed using RIPA buffer [25 mM Tris-HCl, 150 mM NaCl, 1% (v/v) NP-40, 0.5% (w/v) deoxycholate, 0.1% (w/v) SDS, 1 mM Na_3_VO_4_, 1 mM phenylmethylsulfonyl fluoride (PMSF), 10 μg/ml leupeptin] as described previously [31]. Lysates were then ultracentrifuged (185,000 ×g, 30 min, 4°C) and resuspended in the same volume of SDS-PAGE sample buffer, incubated at 95°C for 10 min, then electrophoresed into a 4–12% gradient SDS-PAGE gel (Thermo Fisher) and transferred onto a nitrocellulose membrane.

Cell fractionation was performed as described by Laarmann and Schmidt [32]. Briefly, HeLa cells were scraped from the 100 mm dishes into PBS containing 1 mM Pefabloc and 10 μg ml^−1^ leupeptin, and then sonicated on ice. The sonicated mix was then ultracentrifuged (108,000 ×g, 30 min, 4°C). The supernatant was isolated as the cytosolic fraction and the pellet was washed with PBS before resuspension in buffer containing 0.1 M Na_2_CO_3_/1 M NaCl (pH 11) and incubated on ice for 30 min. The extracted membrane lysate was then ultracentrifuged as above, resulting in a membrane associated fraction in the supernatant and the integral membrane fraction in the pellet. The pellet was solubilised in 2% (w/v) CHAPS in sonication buffer, and ultracentrifuged again, resulting in the detergent resistant integral membrane fraction in the pellet. All fractions were solubilised in SDS-PAGE sample buffer incubated at 95°C for 10 min, then electrophoresed into a 4–12% gradient SDS-PAGE gel (Thermo Fisher), and transferred onto a nitrocellulose membrane.

For far Western blotting, the membrane was blocked in 5% (w/v) skim milk in TBST [50 mM Tris, pH 7.0, 150 mM NaCl, 0.1% Tween 20] and incubated with 12.5 μg IcsA^53-740^ or IcsA^53-740(Δ138–148)^ in TBST with 5% (w/v) skim milk overnight at 4°C. The membrane was then washed with TBS three times and the interaction between IcsA^53-740^ or IcsA^53-740(Δ138–148)^ and host cell proteins was detected with the anti-IcsA antibody as above.

### N-WASP pull down

For N-WASP pull down experiments, approximately 60 μg mini-N-WASP-GST protein purified as described previously [33] was mixed with either 12.5 μg IcsA^53-740^ or IcsA^53-740(Δ138–148)^, and incubated with 200 μl glutathione Sepharose^TM^ 4B (GE Healthcare) resin overnight at 4°C. IcsA^53-740^ and IcsA^53-740(Δ138–148)^ were mixed with or without GST, incubated with glutathione Sepharose^TM^ 4B (GE Healthcare) resin and served as controls. Resins were washed sequentially with PBS; PBS containing 1 mM DTT; PBS containing 0.1% (v/v) NP-40; and PBS for three times each. Protein was eluted in 50 μl PBS containing 20 mM reduced glutathione.

### IcsA structure prediction

The structure of IcsA passenger was acquired using I-TASSER [34] and analysed using Chimera [35].

## Results

### Purification of IcsA passenger protein and refolding

In order to validate the role of IcsA in *Shigella* adherence *in vitro*, the IcsA passenger domain (53–740) without the previously described unstructured region (741–758) [15] was expressed from a pBAD vector with an N-terminal His×12 tag for purification (S1A Fig). IcsA^53-740^ was purified from urea solubilised inclusion bodies via nickel affinity purification. Fractions containing IcsA^53-740^ were pooled and further purified by size exclusion chromatography (S1B Fig) and refolded via dialysis. This purification strategy has significant advantages including high yields and reduced endogenous degradation of the autotransporter passenger domain, a type of domain family that is notoriously difficult to purify in a stable and soluble state.

Since human neutrophil elastase (hNE) has been reported to specifically target *Shigella* surface virulence factors [36] and has previously been used to assess the conformation and folding of IcsA [9], we conducted hNE digestions on the purified IcsA^53-740^ to assess the success of refolding. The purified IcsA^53-740^ showed several resistant fragments, with sizes of approximately 70 kDa, 60 kD, 40 kDa, 12 kDa and 5 kDa (S1D Fig), suggesting that the protein has a compact structure which is resistant to hNE proteolysis. Heat denaturation at 65°C for 5 min resulted in IcsA^53-740^ becoming susceptible to hNE, with complete digestion into fragments less than 15 kDa within the first 5 min (S1E Fig). In addition, the refolded IcsA^53-740^ was able to interact with mini-N-WASP protein (S2 Fig). Together, these data suggest that the purified IcsA^53-740^ was successfully refolded and was functional after purification from inclusion bodies.

### Adherence of hyper-adhesion *Shigella* mutants is highly IcsA dependent

**Fig 1:**
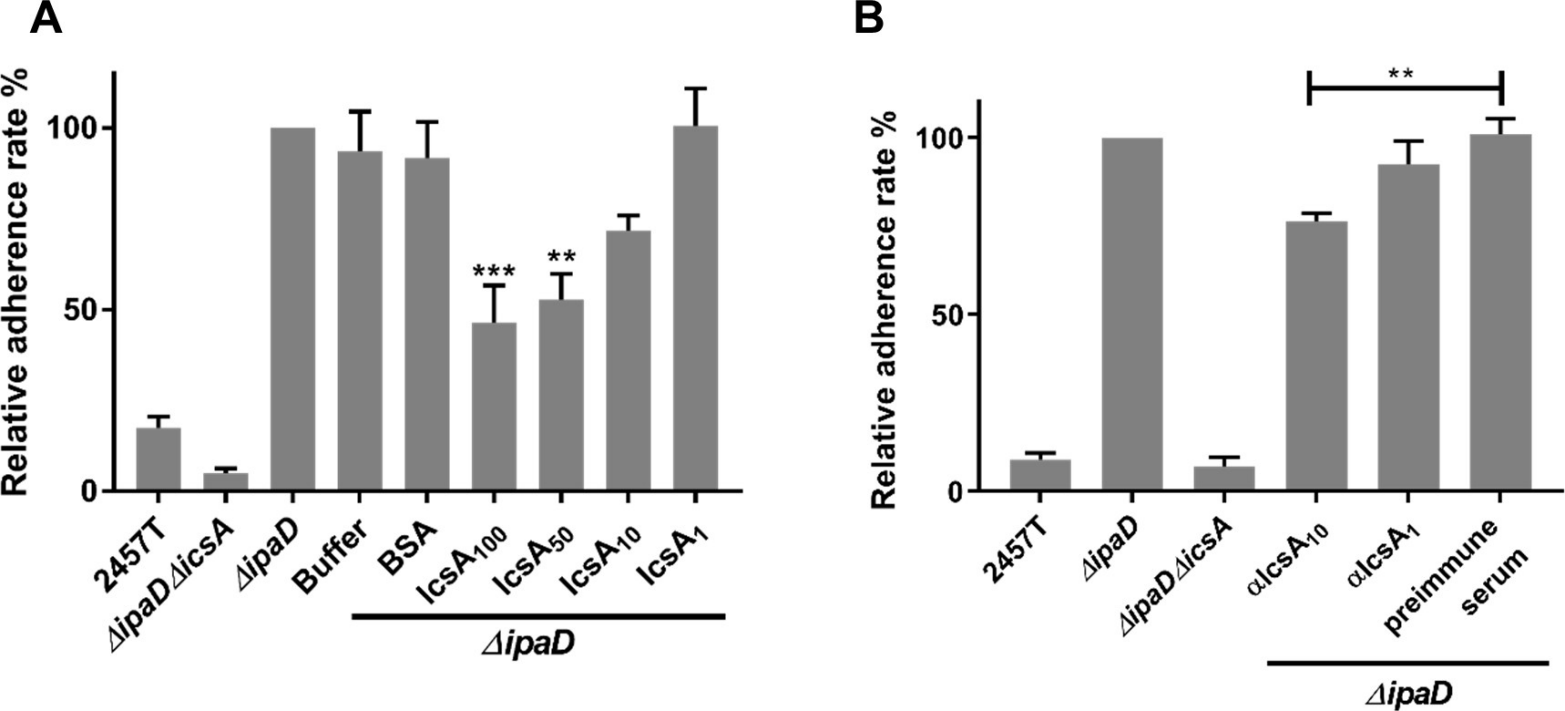
Inhibition of the IcsA-mediated adherence by purified IcsA passenger protein and anti-IcsA antibodies. **A.** IcsA adherence blocking assay. *Shigella* grown to an OD_600_ of 0.5 were collected and used to infect HeLa cell monolayer at the MOI of 100. Purified IcsA^53-740^ protein at the concentration of 2.5 μM (IcsA_100_), 1.25 μM (IcsA_50_), 250 nM (IcsA_10_) and 25 nM (IcsA_1_) were applied at the same time. Refolding buffer and BSA at the concentration of 2.8 μM were used as negative controls. After 15 min incubation, the cell monolayers were washed and lysed. Lysates were serial diluted before dotting on an agar plate for enumeration. Data are normalised against *ΔipaD* (defined as 100%) and are the mean with SEM of three independent experiments. Significance was calculated using one-way ANOVA followed by Dunnett’s multiple comparisons test against *ΔipaD*, and *p* values are as follows: **, *p*<0.01; ***, *p*<0.001. **B.** Antibody adherence blocking assay. *Shigella* grown to an OD_600_ of 0.5 were collected and incubated with 3.125 μg/ml (αIcsA10), 0.3125 μg/ml (αIcsA1) of anti-IcsA antibodies or rabbit pre-immune serum with 100 μg/ml IgG for 15 min before infecting HeLa cell monolayers at the MOI of 100. After an extend 15 min incubation, cell monolayers were treated as in A. Data are normalised against the *ΔipaD* (defined as 100%) and are the mean with SEM of three independent experiments. Significance was calculated using a student *t* test, and *p* values are as follows: **, *p*<0.01.

While previous data strongly indicated that IcsA has adhesin activity [9], this has not been directly demonstrated. We hypothesised that the passenger domain of IcsA directly binds specifically to host cell surface factors in a way that pre-treatment of host cells with purified IcsA^53-740^ would block the adherence of subsequently added *S*. *flexneri*. As expected, an *ΔipaD* mutant strain exhibited an increased adherence phenotype to HeLa cells compared to wild type *S*. *flexneri* (Fig 1A). This increase in adherence is dependent on the presence of IcsA because deletion of IcsA abolished the hyper-adherence (Fig 1A). More importantly, addition of the purified IcsA^53-740^, but not the dialysis buffer or BSA protein, was able to inhibit the adherence of the *ΔipaD* mutant to HeLa cells in a dose dependent manner (Fig 1A). This inhibition was also confirmed for a hyper-adherent *ΔipaB* mutant (S3 Fig). Moreover, these data also confirmed that the purified IcsA^53-740^ protein was folded in a functional conformation. To confirm that endogenous IcsA on the bacterial surface has a direct contribution to the adherence observed for the *ΔipaD* mutant, an adherence blocking assay using polyclonal anti-IcsA antibodies was also conducted (Fig 1B). It was found that pre-treatment of bacteria with anti-IcsA antibodies, but not the rabbit pre-immune serum, was able to significantly block the adherence of the *ΔipaD* mutant to host cells (Fig 1B). Collectively, these data confirmed a direct role of IcsA in the *S*. *flexneri* hyper-adherence activity exhibited by *ΔipaD* and *ΔipaB* mutants.

### IcsA binds specifically to the host cells

**Fig 2:**
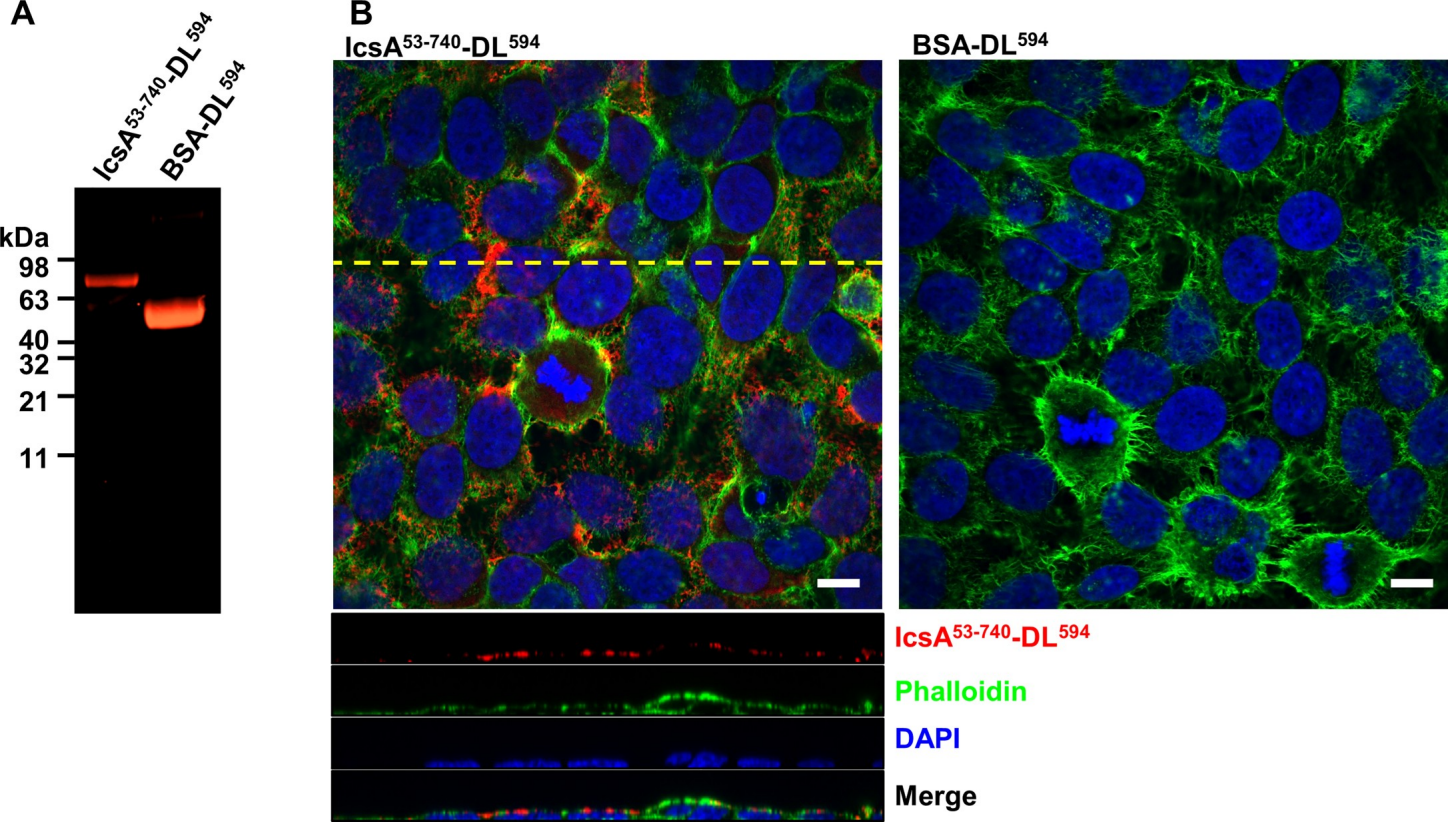
IcsA binds to the surface of HeLa cells. **A.** Fluorescent labelling of IcsA^53-740^. IcsA^53-740^ protein and BSA protein were reacted with DL^594^ maleimide overnight and dialysed against protein solubilisation buffer. Labelled fluorescent protein probes were detected at the 650 nm after SDS-PAGE. **B.** IcsA^53-740^-DL^594^ labelled HeLa cells. IcsA^53-740^-DL^594^ at the concentration of 2.8 μM was applied to cells for 15 min. Samples were then permeabilised and stained with phalloidin and DAPI sequentially. Images were acquired by confocal microscopy with the orthogonal view (position as shown by the dashed yellow line) of a z stack shown below. Cells were stained in the same way with BSA-DL^594^at 28 μM as a negative control. Scale bars = 10 μm.

**Fig 3:**
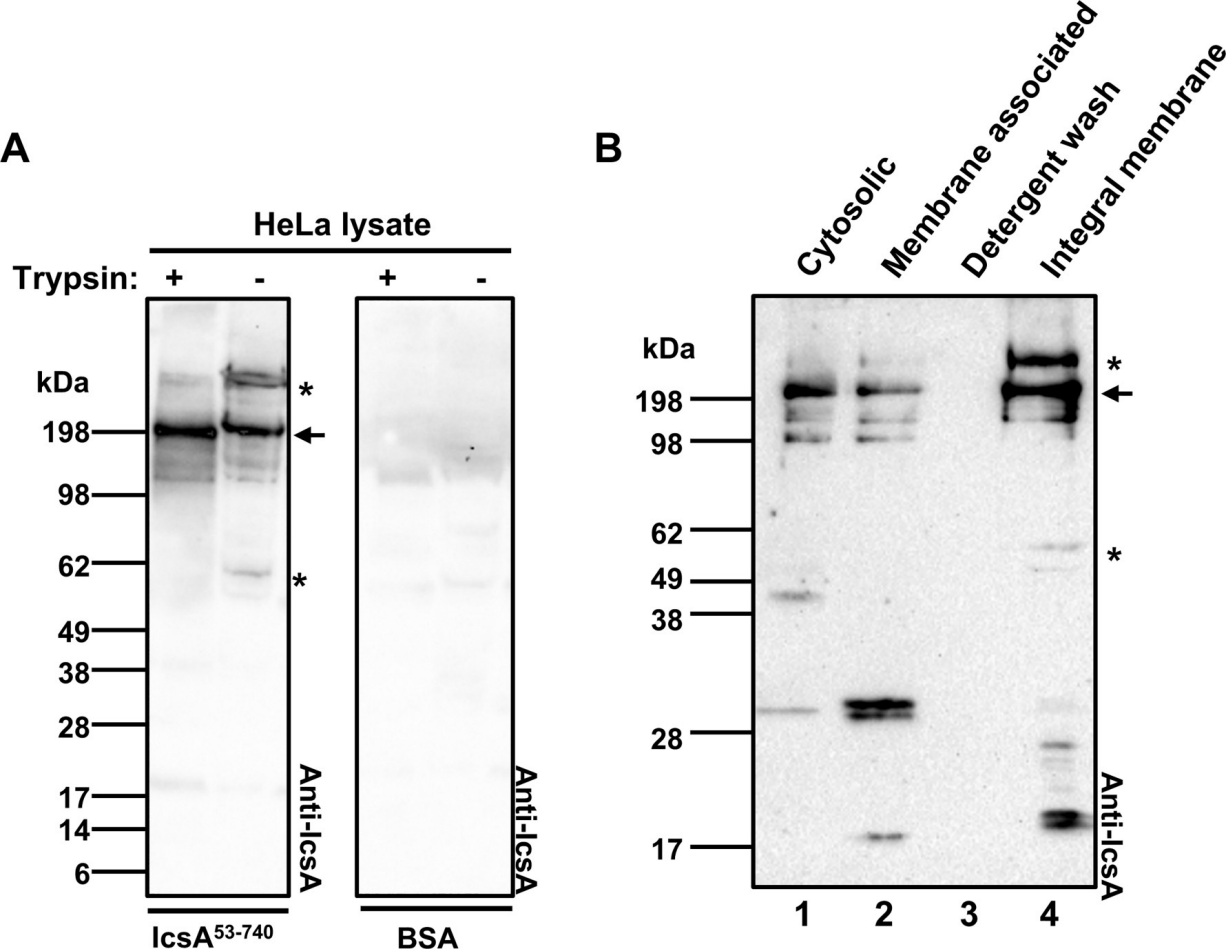
Interactions between IcsA passenger protein and host cell molecules. **A.** Far Western blotting of HeLa cell lysates with IcsA^53-740^. Confluent HeLa cells were recovered either by trypsinisation or cell scraper and lysed by RIPA buffer. Lysates were then separated by SDS-PAGE, transferred onto a nitrocellulose membrane, and probed with IcsA^53-740^ passenger protein or BSA as a negative control. Membranes were subsequently probed with anti-IcsA antibody. **B.** Far Western blotting of HeLa cell fractions with IcsA^53-740^. HeLa cells were lysed and cytosolic and membrane fractions were isolated. All fractions were subjected to far Western blotting, as in A.

Evidence was next generated to determine whether that the IcsA passenger domain binds specifically to host cell molecules as potential receptors for adherence. The purified IcsA^53-740^ was fluorescently labelled by reacting with DL^594^ maleimide (Fig 2A). BSA protein labelled with DL^594^ was used as a control (Fig 2A). Unlike the control BSA- DL^594^, IcsA^53-740^- DL^594^ was detected on the surface of the HeLa cells (Fig 2B). To investigate whether the interaction between IcsA^53-740^ and the HeLa cell surface was specified by a host cell displayed factor, trypsin treated, or untreated HeLa cells lysates were subjected to far Western blotting with IcsA^53-740^ protein (Fig 3A). Two trypsin sensitive molecules (~60 kDa and >200 kDa) were recognised by IcsA^53-740^ protein (Fig 3A). The anti-IcsA antibody showed no cross reaction to the HeLa cell lysate (Fig 3A). To further validate the cell surface location of these IcsA targets, HeLa cells were fractionated (Fig 3B), and subjected to far Western blotting with purified IcsA^53-740^. IcsA interacting components from integral membrane fractions (Fig 3B, lane 4) were detected and two of which were corresponding in size (>200 kDa and ~60 kDa) to the trypsin sensitive molecules from whole cell lysate (Fig 3A). Apart from these two molecules, we also detected bands at approximately 25 kDa and 20 kDa that interacted with IcsA^53-740^ (Fig 3B lane 4). A molecule at ~ 200 kDa was detected across the all fractions (Fig 3B, lane 1, 2 & 4). These data suggest that the interactions between the IcsA^53-740^ passenger domain and the host cell surface is specific and complex.

### IcsA amino acid region 138–148 is required for adhesion

**Fig 4:**
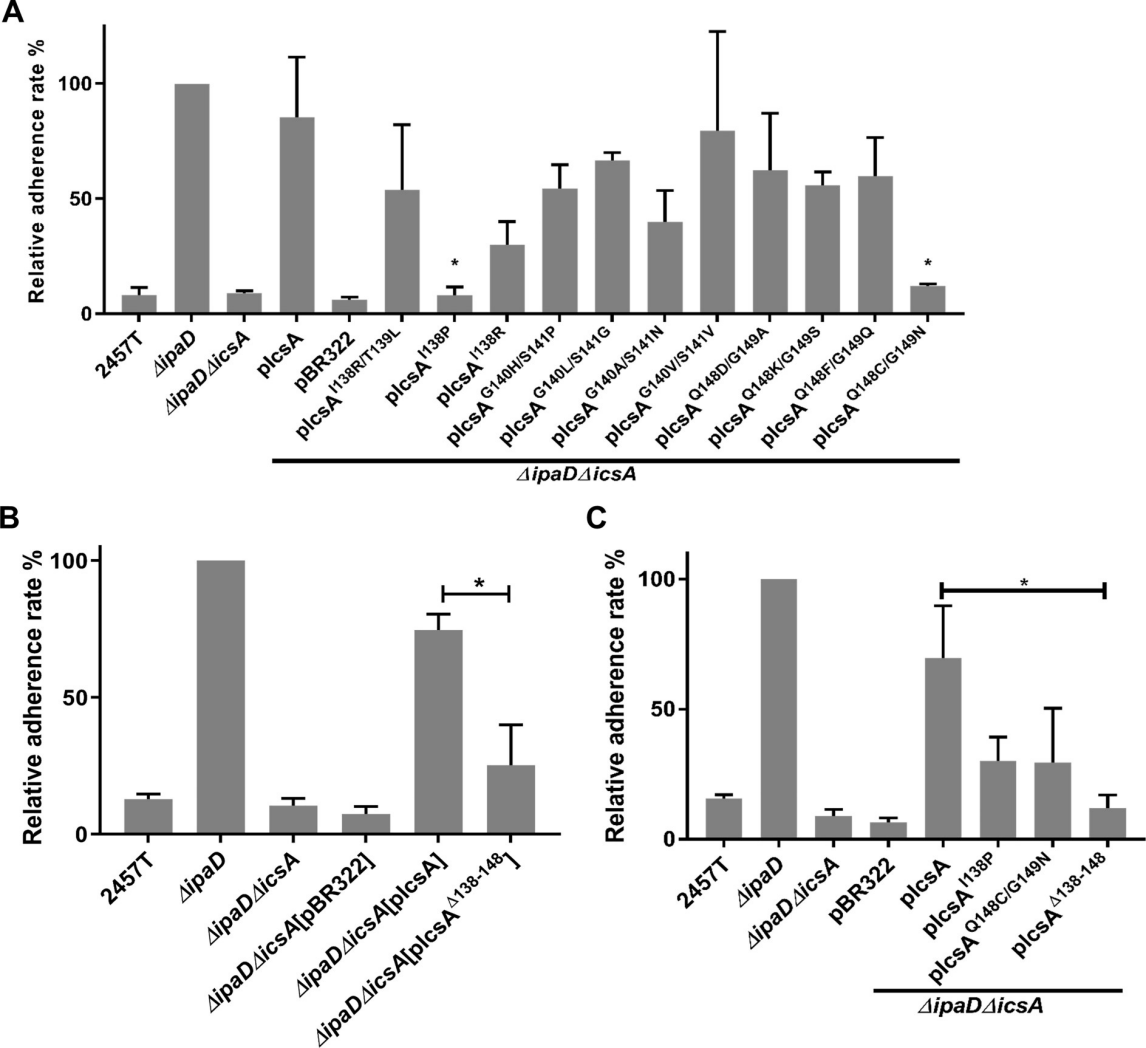
Identification of IcsA adherence function regions. **A.** Screening for adherence related regions using IcsA point mutants and adherence assays. Mid-exponential phase *S*. *flexneri* were collected and used to infect HeLa cell monolayers at a MOI of 100 for 15 min. Monolayers were washed, lysed, and lysates were serial diluted before spotting on an agar plate for enumeration. Data are normalised against *ΔipaD* (defined as 100%) and are the mean with SEM of three independent experiments. Significance was calculated using one-way ANOVA followed by Dunnett’s multiple comparisons test against *ΔipaDΔicsA*[pIcsA], and *p* values are as follows: *, *p*<0.05. **B.** Adherence assay of the IcsA^Δ138–148^ mutant. Significance was calculated using a student *t* test, and *p* values are as follows: *, *p*<0.05. **C.** Adherence assay of IcsA adherent defective mutants. Adherence assays were performed as above with passive settling of bacteria. Significance was calculated using a student *t* test, and *p* values are as follows: *, *p*<0.05.

To identify functional regions required in adhesin activity, we utilised our previously generated plasmid collection that express IcsA mutants harbouring 5 amino acid insertions across the passenger domain [24] to screen for defects in *S*. *flexneri* adherence. These plasmids were introduced into *S*. *flexneri ΔipaDΔicsA* and transformants were used in adherence assays with HeLa cells. In a preliminary experiment attempting to repeat the result of Brotcke-Zumsteg, Goosmann, *et al*. [9], the IcsA^i148^ mutant but not the IcsA^i386^ mutant had an adherence defect (S4A Fig). The screening was then focused on the N-terminus of IcsA passenger domain and three sites (i138, i140 and i148) were found to result in mutated IcsA protein having significant defect in adherence activity (S4B Fig). To further investigate this region (138–148), the amino acids from 138 to 148 were each substituted for alanine and the resulting mutants were screened via HeLa adherence assays (S4C Fig). However, none of these mutants conferred a significant defect in adherence indicating that a larger region, rather than individual residues, drives host receptor interactions. Subsequently, the adjacent amino acids to the i138, i140 and i148 insertion sites (138 and 139, 140 and 141, and 148 and 149 respectively) were randomly substituted and screened for the defects in adherence. Two IcsA mutants (IcsA^I138P^ and IcsA^Q148C/G149N^) were found to cause complete loss of IcsA adherence function (Fig 4A). A deletion spanning this region (IcsA^Δ138–148^), was likewise defective in adherence (Fig 4B). To rule out any confounding effects caused by centrifugation, adherence assays were also performed with passive settling of bacteria (Fig 4C). Compared to the point mutants IcsA^I138P^ and IcsA^Q148C/G149N^, the deletion mutant (IcsA^Δ138–148^) was found to have the greatest defect in adherence (Fig 4C).

The adherence functional region 138 to 148 is within the glycine repeat region [24], thus deletion of this region might affect IcsA biogenesis and/or its ABM function. To test this, the expression, polar localisation, and ABM function of IcsA^Δ138–148^ were confirmed by Western blotting (S5A Fig), immunofluorescent staining (S5B Fig), and plaque formation (S5C Fig) respectively. There was no difference in IcsA expression level, its surface localisation, and the size of plaques formed, between IcsA and IcsA^Δ138–148^. This rules out any major defects in IcsA biogenesis and ABM function for this mutant.

**Fig 5:**
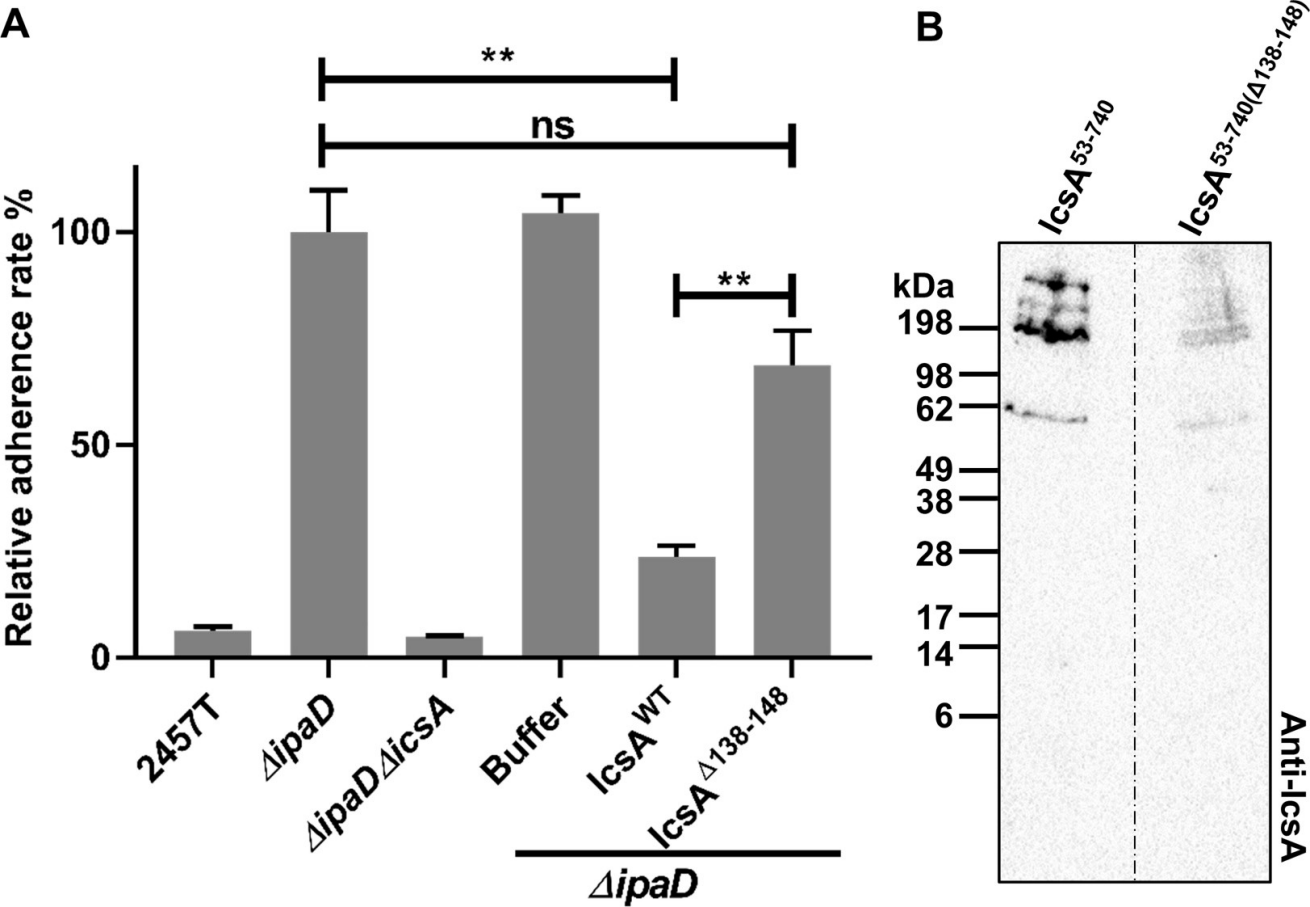
Confirmation of the IcsA adherence related region 138–148. **A.** IcsA adherence blocking assay. *Shigella* grown to an OD_600_ of 0.5 were collected and used to infect HeLa cell monolayer at the MOI of 100. Purified wild type (IcsA^53-740^) or mutant (IcsA^53-740(Δ138–148)^) IcsA passenger protein at the concentration of 1.25 μM were applied at the same time. Refolding buffer was used as a negative control. After 15 min incubation, the cell monolayers were washed and lysed. Lysates were serial diluted before dotting on an agar plate for enumeration. Data are normalised against the mean of *ΔipaD* (defined as 100%) and are the mean with SEM of three independent experiments. Significance was calculated using a student *t* test, and *p* values are as follows: **, *p*<0.01. **B.** Far Western blotting of HeLa cell lysates with wild type (IcsA^53-740^) or mutant (IcsA^53-740(Δ138–148)^) IcsA passenger protein. HeLa cells grown on 100 mm dish were recovered either by trypsin or cell scraper, and lysed by RIPA buffer. Lysates were then separated by 12% SDS-PAGE, transferred onto a nitrocellulose membrane, and probed by either IcsA^53-740^ or mutant IcsA^53-740(Δ138–148)^ protein (12.5 μg) overnight at 4°C. The membrane was then washed by TBST and subjected to Western blotting with anti-IcsA antibody. Note that B contains two membranes (indicated by the dashed line) that were incubated with antibodies and imaged together.

**Fig 6:**
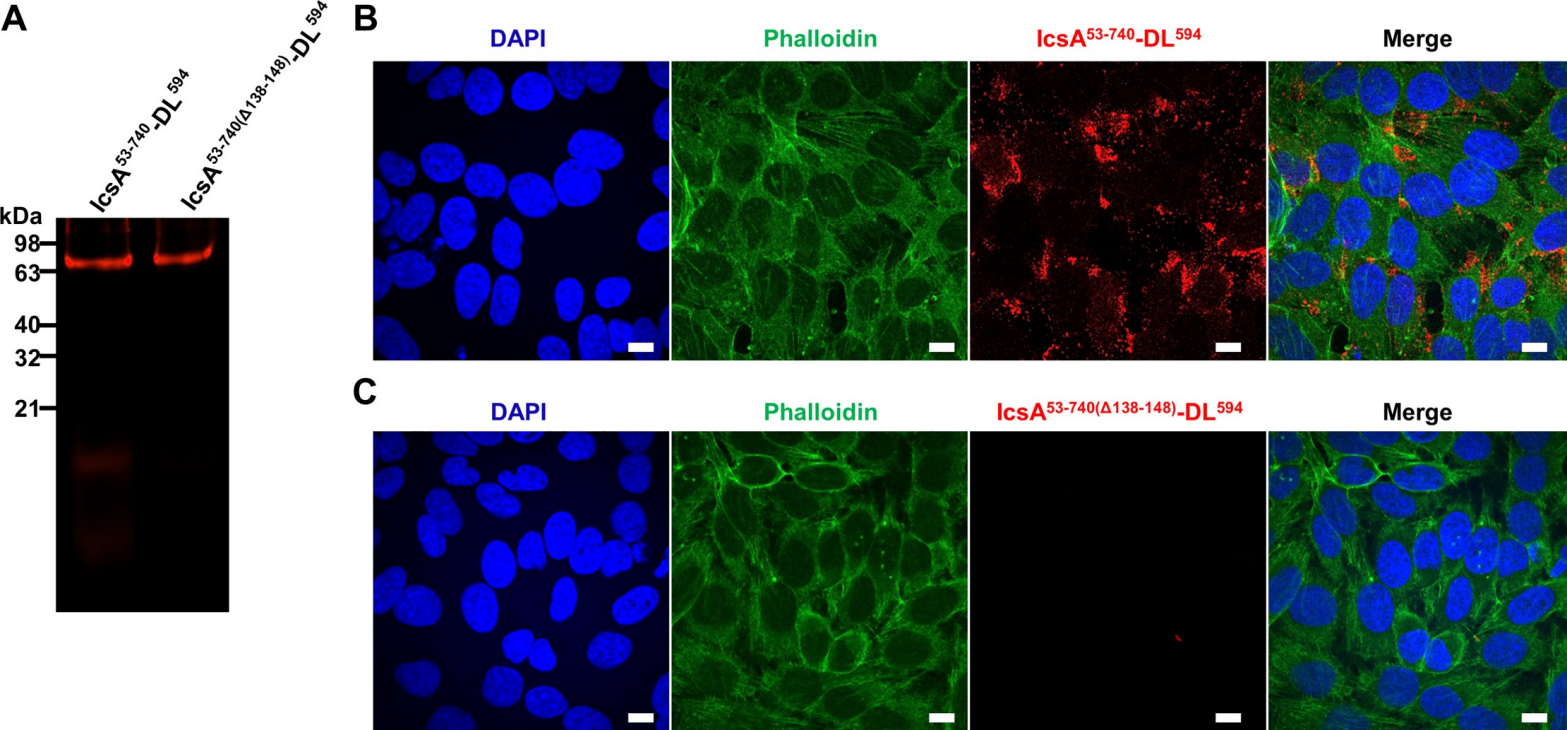
IcsA^Δ138–148^ is defective in binding to HeLa cells. **A.** Fluorescent labelling of IcsA^53-740^ and IcsA^53-740(Δ138–148)^. IcsA^53-740^and IcsA^53-740(Δ138–148)^ were reacted with DL^594^ maleimide overnight and dialysed against protein solubilisation buffer. Labelled fluorescent protein probes were detected at the 650 nm after SDS-PAGE. **B.** IcsA^53-740^ protein labelled HeLa cells. IcsA^53-740^-DL^594^ at the concentration of 1.5 μM was applied in the assay. After an incubation of 15 min with HeLa monolayers, samples were then permeabilised and stained with phalloidin and DAPI sequentially. Image was acquired by confocal microscopy. **C.** IcsA^53-740(Δ138–148)^ protein labelled HeLa cells. IcsA^53-740(Δ138–148)^-DL^594^ at the concentration of 1.5 μM was applied in the assay and samples were treated the same as in A. Scale bars = 10 μm.

To validate that IcsA^Δ138–148^ has a defect in adherence *in vitro*, IcsA^53-740(Δ138–148)^ was expressed, purified, and refolded in an equivalent manner to IcsA^53-740^. Refolded IcsA^53-740(Δ138–148)^ was able to interact with mini-N-WASP protein *in vitro* (S2 Fig), confirming that the region 138–148 is not essential for IcsA’s ABM function, and that purified IcsA^53-740(Δ138–148)^ protein was functional. However, relative to IcsA^53-740^, IcsA^53-740(Δ138–148)^ was unable to block the adherence of *S*. *flexneri ΔipaD* (Fig 5A). Moreover, in far Western blotting of HeLa cell lysates, unlike IcsA^53-740^, IcsA^53-740(Δ138–148)^ had greatly reduced interaction with host molecules (Fig 5B), given that the deletion of 138–148 did not affect the recognition via anti-IcsA antibody (S2 Fig). Fluorescently labelled IcsA^53-740(Δ138–148)^ (Fig 6A) was prepared and used to label HeLa cells, but no staining was detected (Fig 6C). These data further supporting the notion that residues 138 to 148 affect IcsA’s adhesin function.

**Fig 7:**
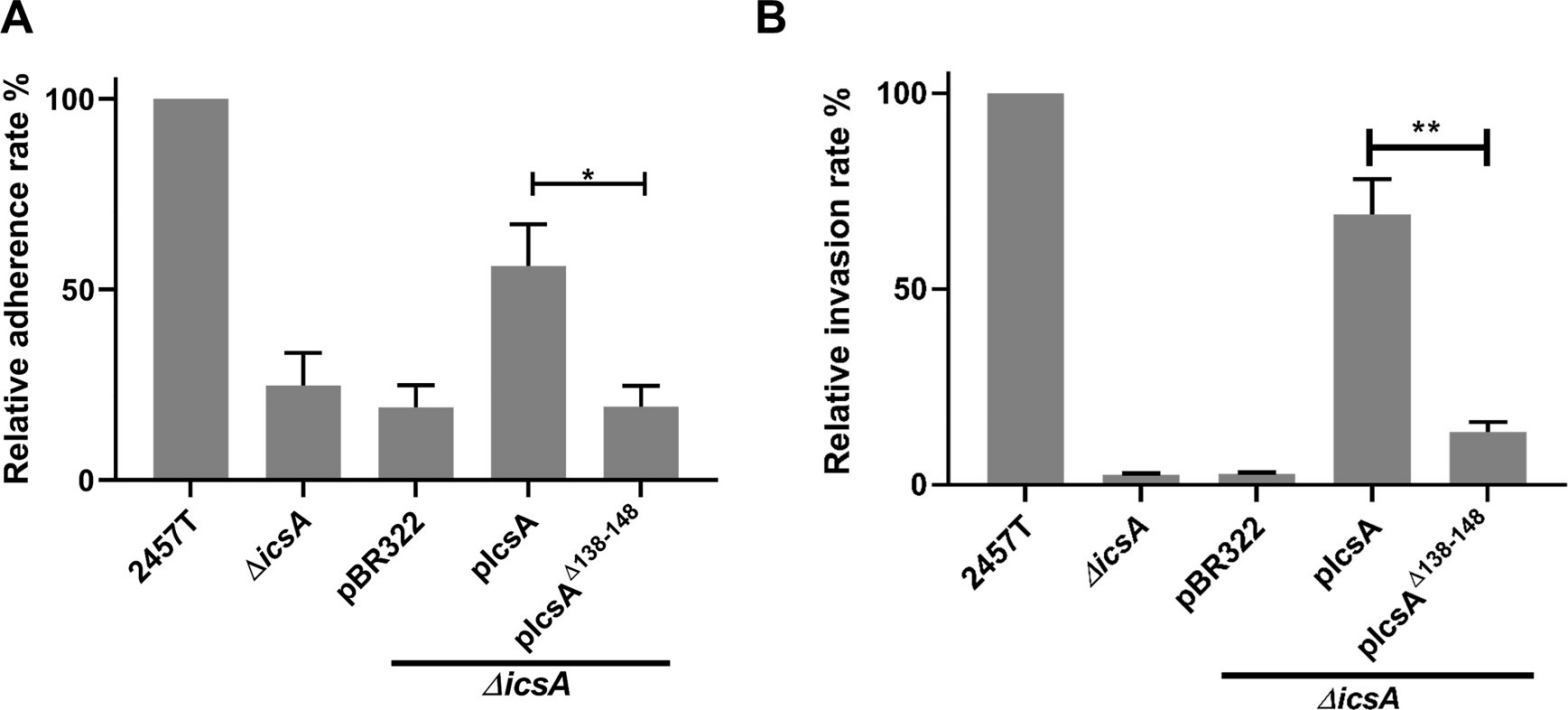
The adherence region IcsA 138–148 is required for *Shigella* adhesion and invasion during DOC stimulation. **A.** Adherence assay. *Shigella* grown to an OD_600_ of 0.5 in the presence of 2.5 mM DOC were collected and used to infect HeLa cell monolayer at the MOI of 100. After 15 min incubation, the cell monolayers were washed and lysed. Lysates were serial diluted before dotting on an agar plate for enumeration. Data are normalised against 2457T (defined as 100%) and are the mean with SEM of three independent experiments. **B.** Invasion assay. *Shigella* grown to an OD_600_ of 0.5 in the presence of 2.5 mM DOC were collected and used to infect HeLa cell monolayer at the MOI of 100. At the 45 min post-infection, gentamicin was added and incubated with the cell monolayers for another 45 min. Cell monolayers were then treated as in A. Significance was calculated using a student *t* test, and *p* values are as follows: *, *p*<0.05; **, *p*<0.01.

In the human gut, *Shigella* virulence is activated by, among other stimuli, bile salt components such as DOC [37].The impact of the aa 138–148 region on DOC induced hyper-adherence was investigated. As expected, DOC at the physiological concentration 2.5 mM was able to enhance the adherence of *Shigella* significantly (Fig 7A). However, the IcsA^Δ138–148^ mutant displayed a significant defect in the DOC enhanced adherence (Fig 7A), which again confirmed that the region from 138–148 is required for the IcsA-mediated adherence. Moreover, invasion of HeLa cells by *S*. *flexneri ΔicsA* complemented with IcsA^Δ138–148^ to HeLa cells was also significantly attenuated (Fig 7B), indicating that the IcsA-mediated adherence is required for *Shigella* invasion.

## Discussion

In this work we have generated evidence that IcsA directly contributes to adherence of *Shigella* species to host cells. We were able to block the hyper-adherence phenotype of *S*. *flexneri ΔipaD* strains using either purified IcsA^53-740^ passenger domain or anti-IcsA antibody. Purified IcsA^53-740^ was able to bind host cell surfaces and recognised a multitude of host cell molecules. In addition, IcsA residues 138–148 were shown to be critical for adhesin function and purified IcsA^53-740(Δ138–148)^ could no longer block *Shigella* adherence to host cells and was unable to recognise host cell molecules via far Western immunoblotting.

IcsA does not have detectable adherence activity in wild-type *S*. *flexneri* unless exposed to environmental stimuli (such as DOC) [37], or via activation of the T3SS [9]. IcsA adherence activity is strongly associated to a conformational change as detected by proteinase accessibility [9]. Nevertheless, in *E*. *coli*, heterogeneously expressed IcsA can promote bacterial adherence to host cells [9], presumably because the conformation, stimuli, folding, or modification of IcsA is different to that of the *S*. *flexneri*. Indeed, through hNE digestion analysis on our refolded IcsA^53-740^, we detected a resistant fragment of ~40 kD similarly to that reported previously for the hNE digestion of IcsA on intact *S*. *flexneri* bacteria [9], where a fragment around 40 kDa was more resistant to degradation by hNE in those strains with increased IcsA-mediated adherence. It is plausible that in *Shigella*, IcsA’s function in adherence is carefully downregulated by some mechanism governed by the T3SS before it encounters an environmental cue, such as DOC, whereas in *E*. *coli*, lack of such a regulating system allows IcsA to exert its adherence function constitutively.

The purified and refolded IcsA^53-740^ passenger domain retains its activity as a *Shigella* adhesin. Due to this, pre-incubation of the HeLa cells with purified IcsA^53-740^protein blocks the adherence of *S*. *flexneri ΔipaD* and *ΔipaB* strain. The minimum IcsA concentration in our experiments showing significant adherence blocking was 1.25 μM, which is approximately 10,000 times to the IcsA molecules expressed per input bacteria, assuming that each bacterium expresses approximately 4,000 IcsA molecules on the surface [38]. This is likely because purified IcsA^53-740^ must bind to many host cell receptors to block adherence. Indeed, comparable concentrations of antigens were also used to block virus entry [39] and bacteria adherence [40] to host cells. This is also supported by both the fluorescent labelling of HeLa cell surface and the far Western immunoblotting with purified IcsA^53-740^, indicating a specific and complex interaction between IcsA and host cells. It was not surprising that IcsA^53-740^ recognised other molecules from both cytosolic and membrane associated fractions, as IcsA is known to interact with cytosolic molecules responsible for actin based motility [19] and is recognised by host cell autophagic systems [41]. In our antibody blocking assay, the anti-IcsA antibody at 3.125 μg/ml blocked the *Shigella* adherence significantly, which is comparable to the concentrations of antibodies used in other studies [42–44]. Given that our data support a model where IcsA may recognise multiple receptors on host cells, and that anti-IcsA antibodies were able to neutralise *Shigella* adherence *in vitro*, this strongly suggests that the IcsA passenger domain has excellent vaccine potential.

The previous study using our IcsA insertion library found insertions at sites 148 and 386 affected IcsA-mediated *Shigella* adherence [9]. However, in the present study i138, i140, and i148, but not i386, were found to result in decreased IcsA-mediated adherence. The discrepancy at the site 386 is possibly due to the differences in the screening system. In the previous study [9], IcsA mutants with normal ABM function and defects in DOC-enhanced *Shigella* invasion were selected, whereas in this study, the adherence region was screened directly by assessing the adherence of *Shigella* IcsA insertion mutants to HeLa cells. While alanine scanning mutagenesis of the region 138–148 showed no significant defect in adherence, it is likely that the entire region 138–148 is required for multiple contacts between IcsA and host cell receptors such that the overall interface stability between IcsA and receptor cannot be significantly reduced by any single alanine substitution. Nevertheless, site-directed mutagenesis on the IcsAi-adjacent amino acids revealed substitution mutants (I138P and Q148C/G149N) that resulted in significant defects in adherence. Moreover, deletion of the entire region (138–148) in the IcsA passenger domain also reproduced the defect of the 5aa insertions, and was much more severe compared to the double mutants.

We speculate that this 138–148 IcsA region is involved in host cell receptor binding events. Indeed, in a predicted IcsA passenger structure (S6 Fig), the predicted β-rung harbouring aa 138–148 is smaller than the adjacent β-helixes and generates a groove in the IcsA passenger domain that might function as a putative receptor binding cleft. The Q148C found next to another cysteine (C130) in space located on the adjacent β-strand in the β-helix potentially allows the formation of a disulfide bond that might obstruct this binding cleft. Furthermore, purified mutant IcsA^53-740(Δ138–148)^ passenger domain was unable to block adherence and showed reduced interactions to host cell components further supporting our speculations that this region might be a binding cleft for host receptors. Even DOC-stimulated hyper-adherence of *S*. *flexneri* expressing mutant IcsA (IcsA^Δ138–148^) consistently showed a defect in adherence and host cell invasion. IcsA has established functional roles in binding of host cell cytosolic factors to nucleate ABM, and the IcsA passenger domain can also accommodate further functions in adhesion via a specific binding region. Therefore, IcsA is a truly multifunctional virulence factor providing an avenue for *Shigella* to quickly respond to the pathogenic niche for adhesion, invasion, and spreading [45].

## Supporting information

S1 Fig Expression, purification and refolding of the IcsA passenger protein. **A.** Schematic representation of IcsA passenger expression construct. IcsA passenger from amino acid 53 to 740 was fused with a His_12_ tag; its expression in *E*. *coli* Top10 was controlled by the pBAD promoter. EK, enterokinase site. **B.** Coomassie blue staining of purified fractions containing IcsA^53-740^ protein. IcsA^53-740^ protein (indicated by the arrow) was solubilised from inclusion bodies, purified through nickel affinity chromatography and further cleaned by size exclusion gel filtration. Peak fractions were analysed by SDS-PAGE and stained by Coomassie blue. **C.** IcsA passenger refolding buffer screening. IcsA^53-740^protein was diluted 1 in 20 into different buffer solutions (as indicated), and after an incubation of approximately 16 h at 4°C, solutions were ultracentrifuged, resulting in the soluble fractions in the supernatant (S) and the insoluble fractions in the aggregates (Ag). Both fractions were separated by 12% SDS-PAGE and transferred onto nitrocellulose membrane and stained with Ponceau S. Buffer solutions are all based on 50 mM NaCl, 50 mM Tris, pH 8, unless where stated. **D.** Limited proteolysis of refolded IcsA^53-740^ protein by human neutrophil elastase (hNE). Following purification, IcsA^53-740^ protein was dialysed and digested by hNE in the molecular ratio of 1000:1. Sample from different time points were taken and analysed by Coomassie blue stained SDS-polyacrylamide gel. **E.** Limited proteolysis of heat inactivated IcsA^53-740^ protein by human neutrophil elastase (hNE). Refolded IcsA^53-740^ protein was heated to 65°C for 15 min and cooled to room temperature before being digested by hNE in the molecular ratio of 1000:1.
(TIF)

S2 Fig Purified IcsA protein was able to interact with mini-N-WASP. IcsA^53-740^ and IcsA^53-740(Δ138–148)^ were mixed with mini-N-WASP-GST, incubated with glutathione resin overnight. IcsA^53-740^ and IcsA^53-740(Δ138–148)^ were mixed with or without GST, incubated with glutathione resin and served as controls. Resin was then washed, and protein was eluted and analysed via a 12% SDS-PAGE gel and Western immunoblotting using anti-IcsA antibody (upper) **or** anti-GST antibody (lower).
(TIF)

S3 Fig Inhibition of the IcsA-mediated adherence of *S. flexneri ΔipaB* with IcsA^53-740^ protein. *Shigella* grown to an OD_600_ of 0.5 were collected and used to infect HeLa cell monolayer at the MOI of 100. Purified IcsA^53-740^ protein at the concentration of 2.5 μM (IcsA_100_), 1.25 μM (IcsA_50_), 250 nM (IcsA_10_) and 25 nM (IcsA_1_) were applied at the same time. Refolding buffer and BSA at the concentration of 2.8 μM were used as negative controls. After 15 min incubation, the cell monolayers were washed and lysed. Lysates were serial diluted before dotting onto agar plates for enumeration. Data are normalised against the mean of *ΔipaB* (defined as 100%) and are the mean with SEM of four independent experiments. Significance was calculated using one-way ANOVA followed by Dunnett’s multiple comparisons test against *ΔipaB*, and *p* values are as follows: ****, *p*<0.0001.
(TIF)

S4 Fig Screening of the IcsA mutants via adherence assays. **A.** Screening of the putative adherence defective IcsA mutants via adherence assay. *Shigella ΔipaDΔicsA* expressing the indicated IcsA mutant constructs were grown to an OD_600_ of 0.5 and used to infect HeLa cell monolayer at the MOI of 100. After 15 min infection, the cell monolayers were washed and lysed. Lysates were serial diluted before dotting onto agar plates for enumeration. Data are normalised against *ΔipaD* (defined as 100%) and are the mean with SEM of three independent experiments. Significance was calculated using a student *t* test, and *p* values are as follows: *, *p*<0.05. **B.** Screening of the *Shigella* IcsA 5aa insertion mutants via adherence assays performed as in **A**. Data represent two independent experiments. Significance was calculated using one-way ANOVA followed by Dunnett’s multiple comparisons test against *ΔipaDΔicsA*[pIcsA], and *p* values are as follows: **, *p*<0.01. **C.** Alanine scanning of the IcsA adherent region via adherence assays. *Shigella ΔipaDΔicsA* expressing the indicated IcsA mutant constructs were used to infect HeLa cells as in A. Data represent two independent experiments. Experiments and statistical analysis were performed as above. ns: non-significant.
(TIF)

S5 Fig The region 138–148 does not affect IcsA’s expression, localization and ABM function. **A.** Western immunoblotting of *S*. *flexneri* 2457T, and *ΔicsA* expressing IcsA or IcsA^Δ138–148^. *Shigella* strains grown to an OD_600_ of 0.5 were collected and analysed via a 12% SDS-PAGE gel and Western immunoblotting with anti-IcsA. **B.** Immunofluorescent staining of IcsA with whole *Shigella* bacteria. Bacteria grown to an OD_600_ of 0.5 were collected and fixed with formaldehyde. IcsA was stained with rabbit anti-IcsA, and Alexa Fluor 488 conjugated donkey anti-rabbit antibodies. Images were acquired using an Olympus epifluorescence microscope [24]. Scale bar represents 2 μm. **C.** Plaque formation assay with IcsA mutants and their complemented strains. *Shigella* grown to an OD_600_ of 0.5 were collected to infect HeLa cell monolayers. After 1.5 h infection, the extracellular bacteria was killed by adding DMEM supplemented with 0.5% (w/v) agar and 40 μg/ml gentamycin. After 24 h post-infection, a second layer of DMEM medium containing 0.5% (w/v) agar and 0.1% (w/v) Neutral Red was added and images were taken after 72 h post-infection. **D.** Plaque size measurements for plaques formed in **C**. Data were acquired at least from 20 plaques for each strain and significance was calculated using a student *t* test, and p values are as follow: ns, non-significant. Note that *ΔicsA* and *ΔicsA* [pBR322] did not form plaques.
(TIF)

S6 Fig Structural analysis of the amino group substitution sites in the IcsA passenger domain. **A.** Predicted structure of IcsA passenger 55–241 shown in ribbon. **B.** Side view of the ribbon structure of IcsA^55-241^. **C.** Surface of the predicted IcsA^55-241^ structure. The structure of the IcsA passenger (55–241) was acquired from Itasser and annotated using Chimera. The amino group adjacent to the insertion sites (i138, i140 and i148) are marked on the structure. The IcsA adherent region is shown in green.
(TIF)

S1 Raw Images Raw image files of Western immunoblotting and SDS-PAGE gels in the manuscript. (PDF)

S1 Table Strains and plasmids. (PDF)

S2 Table Oligonucleotides. (PDF)
