# Supplementary figures and images for: The virulence domain of Shigella IcsA contains a subregion with specific host cell adhesion function
Source: PLoS One. 2020 Jan 7;15(1):e0227425. doi: 10.1371/journal.pone.0227425 (PMC6946128; doi:10.1371/journal.pone.0227425)

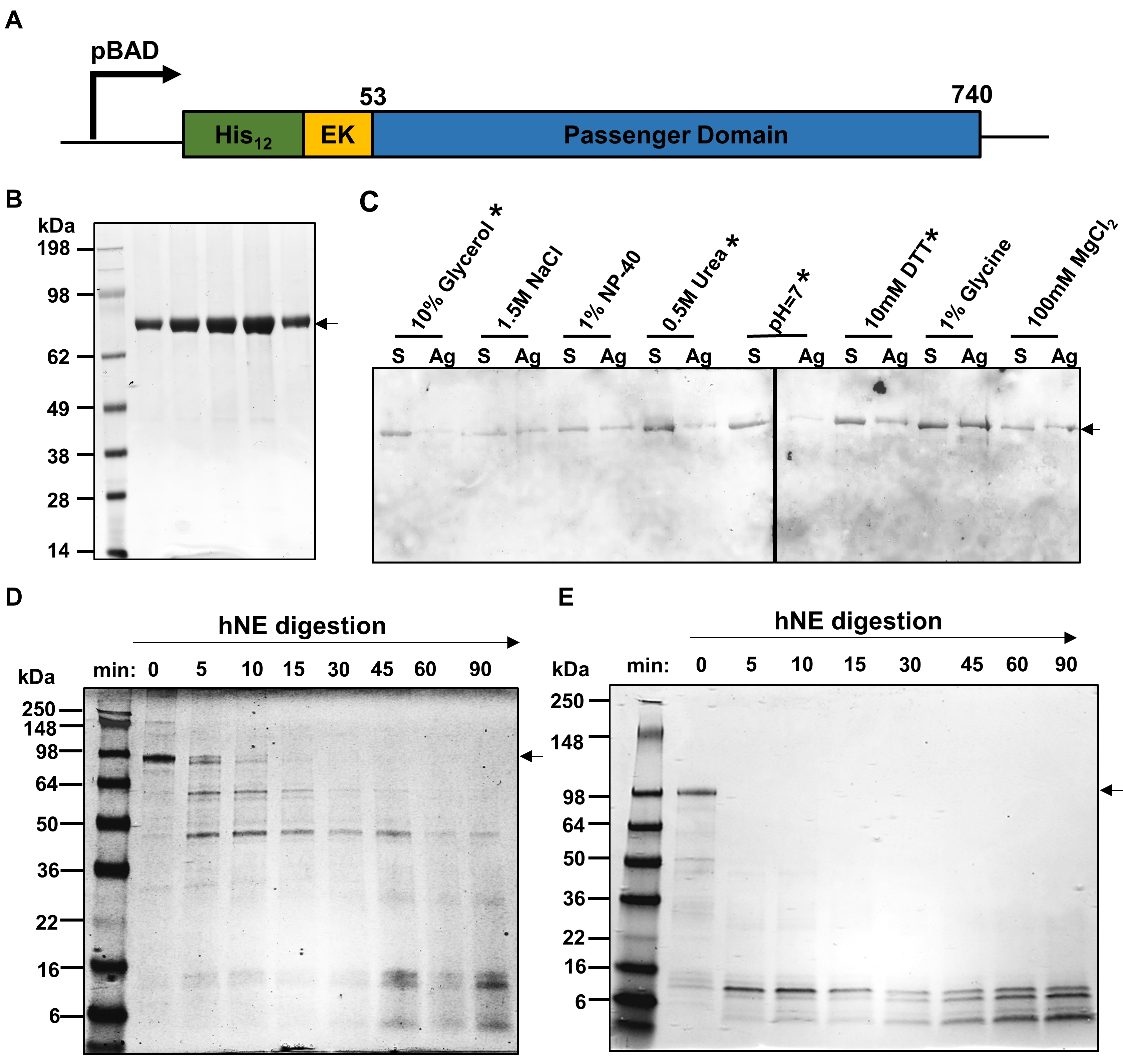

Supplement: S1 Fig — A. Schematic representation of IcsA passenger expression construct. IcsA passenger from amino acid 53 to 740 was fused with a His12 tag; its expression in E. coli Top10 was controlled by the pBAD promoter. EK, enterokinase site. B. Coomassie blue staining of purified fractions containing IcsA53-740 protein. IcsA53-740 protein (indicated by the arrow) was solubilised from inclusion bodies, purified through nickel affinity chromatography and further cleaned by size exclusion gel filtration. Peak fractions were analysed by SDS-PAGE and stained by Coomassie blue. C. IcsA passenger refolding buffer screening. IcsA53-740protein was diluted 1 in 20 into different buffer solutions (as indicated), and after an incubation of approximately 16 h at 4°C, solutions were ultracentrifuged, resulting in the soluble fractions in the supernatant (S) and the insoluble fractions in the aggregates (Ag). Both fractions were separated by 12% SDS-PAGE and transferred onto nitrocellulose membrane and stained with Ponceau S. Buffer solutions are all based on 50 mM NaCl, 50 mM Tris, pH 8, unless where stated. D. Limited proteolysis of refolded IcsA53-740 protein by human neutrophil elastase (hNE). Following purification, IcsA53-740 protein was dialysed and digested by hNE in the molecular ratio of 1000:1. Sample from different time points were taken and analysed by Coomassie blue stained SDS-polyacrylamide gel. E. Limited proteolysis of heat inactivated IcsA53-740 protein by human neutrophil elastase (hNE). Refolded IcsA53-740 protein was heated to 65°C for 15 min and cooled to room temperature before being digested by hNE in the molecular ratio of 1000:1. (TIF) [file pone.0227425.s001.tif]

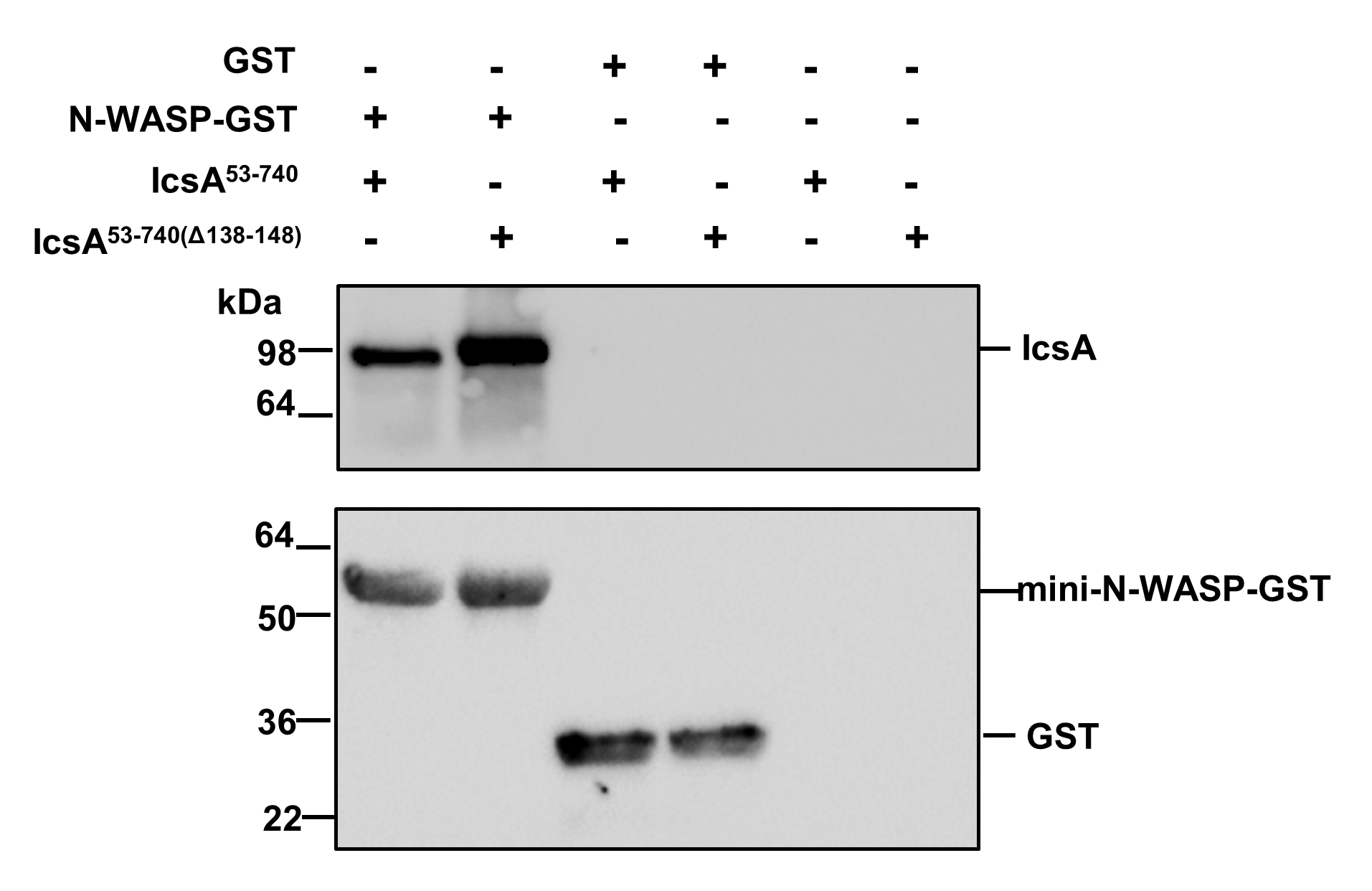

Supplement: S2 Fig — IcsA53-740 and IcsA53-740(Δ138–148) were mixed with mini-N-WASP-GST, incubated with glutathione resin overnight. IcsA53-740 and IcsA53-740(Δ138–148) were mixed with or without GST, incubated with glutathione resin and served as controls. Resin was then washed, and protein was eluted and analysed via a 12% SDS-PAGE gel and Western immunoblotting using anti-IcsA antibody (upper) or anti-GST antibody (lower). (TIF) [file pone.0227425.s002.tif]

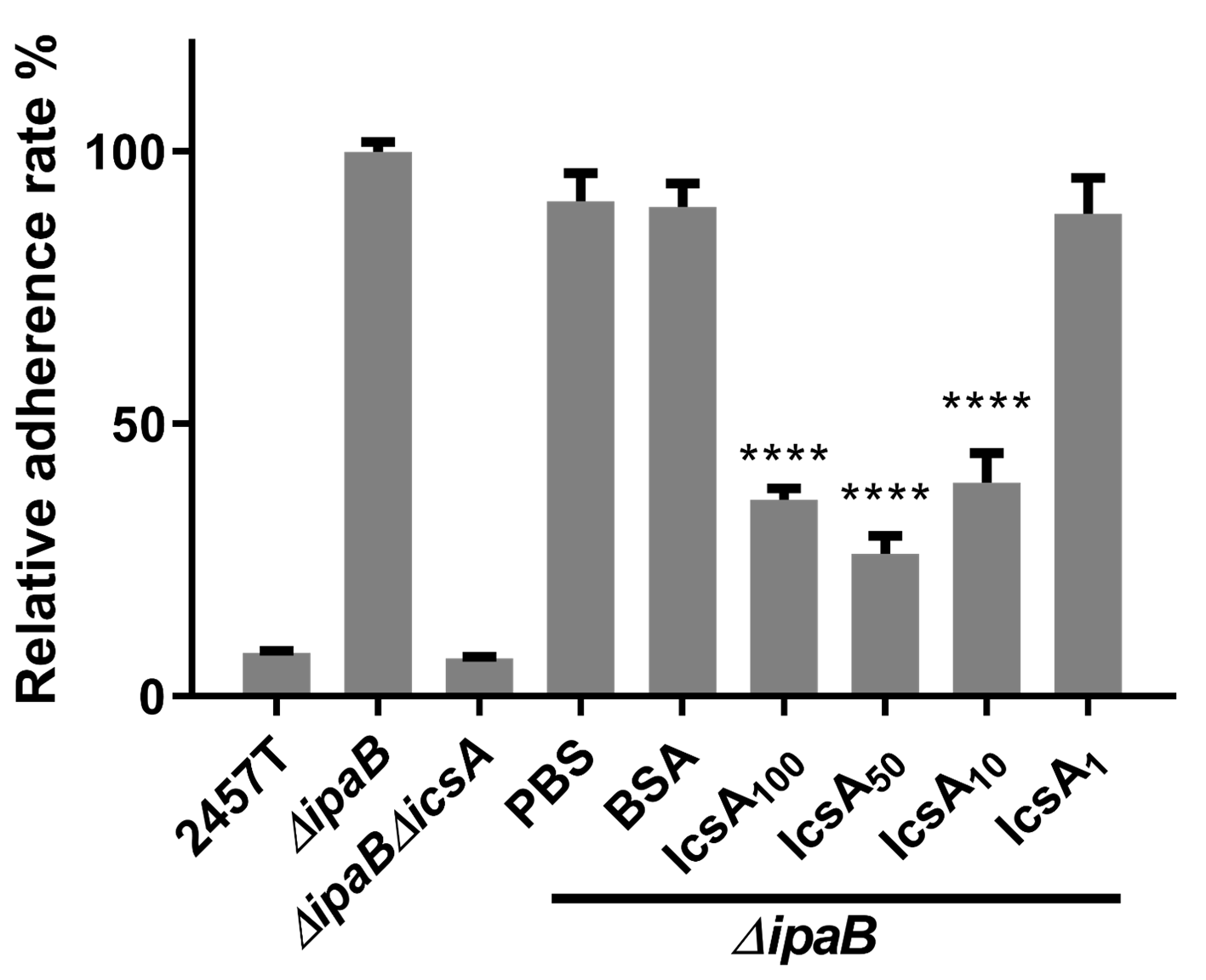

Supplement: S3 Fig — Shigella grown to an OD600 of 0.5 were collected and used to infect HeLa cell monolayer at the MOI of 100. Purified IcsA53-740 protein at the concentration of 2.5 μM (IcsA100), 1.25 μM (IcsA50), 250 nM (IcsA10) and 25 nM (IcsA1) were applied at the same time. Refolding buffer and BSA at the concentration of 2.8 μM were used as negative controls. After 15 min incubation, the cell monolayers were washed and lysed. Lysates were serial diluted before dotting onto agar plates for enumeration. Data are normalised against the mean of ΔipaB (defined as 100%) and are the mean with SEM of four independent experiments. Significance was calculated using one-way ANOVA followed by Dunnett’s multiple comparisons test against ΔipaB, and p values are as follows: ****, p<0.0001. (TIF) [file pone.0227425.s003.tif]

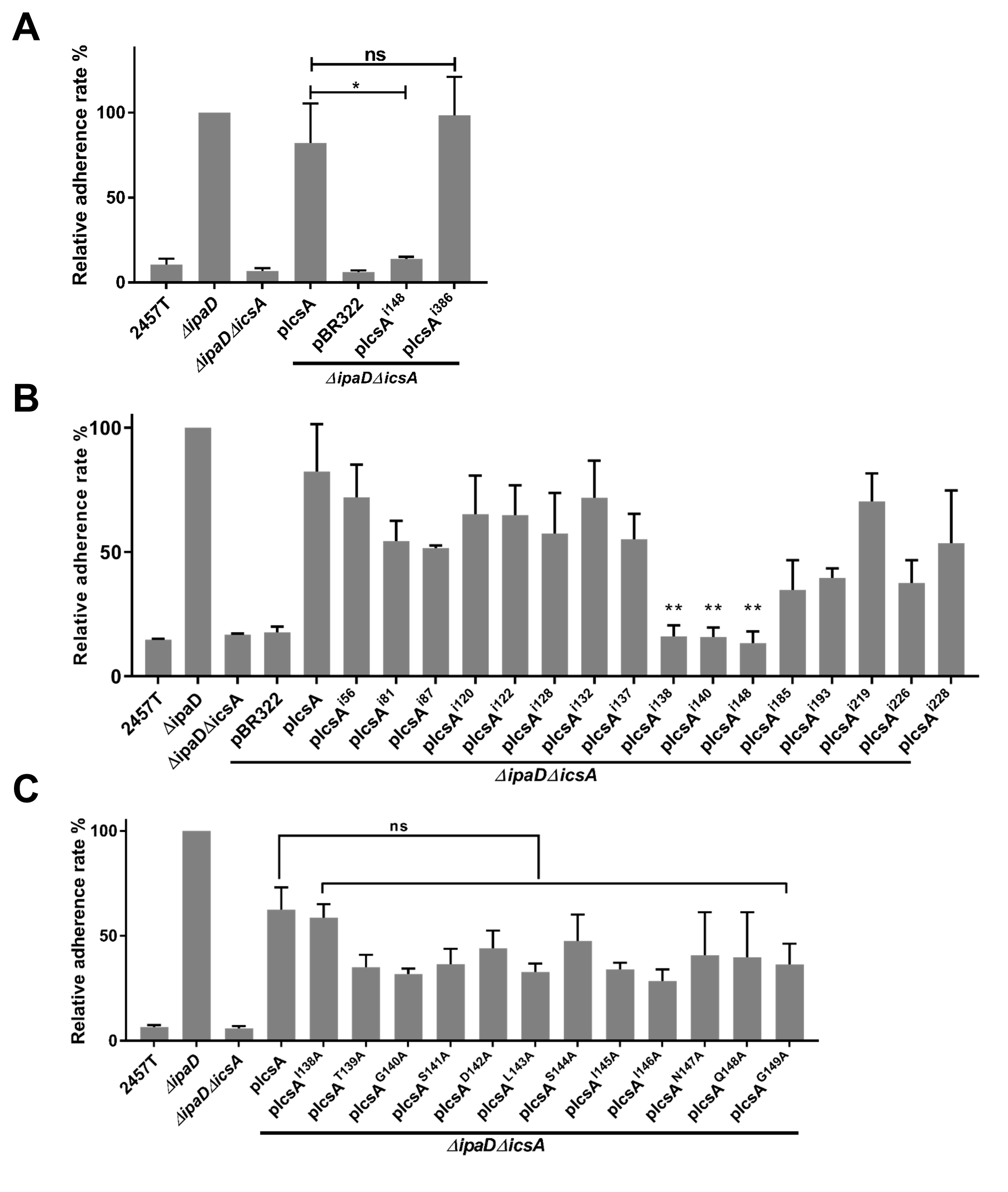

Supplement: S4 Fig — A. Screening of the putative adherence defective IcsA mutants via adherence assay. Shigella ΔipaDΔicsA expressing the indicated IcsA mutant constructs were grown to an OD600 of 0.5 and used to infect HeLa cell monolayer at the MOI of 100. After 15 min infection, the cell monolayers were washed and lysed. Lysates were serial diluted before dotting onto agar plates for enumeration. Data are normalised against ΔipaD (defined as 100%) and are the mean with SEM of three independent experiments. Significance was calculated using a student t test, and p values are as follows: *, p<0.05. B. Screening of the Shigella IcsA 5aa insertion mutants via adherence assays performed as in A. Data represent two independent experiments. Significance was calculated using one-way ANOVA followed by Dunnett’s multiple comparisons test against ΔipaDΔicsA[pIcsA], and p values are as follows: **, p<0.01. C. Alanine scanning of the IcsA adherent region via adherence assays. Shigella ΔipaDΔicsA expressing the indicated IcsA mutant constructs were used to infect HeLa cells as in A. Data represent two independent experiments. Experiments and statistical analysis were performed as above. ns: non-significant. (TIF) [file pone.0227425.s004.tif]

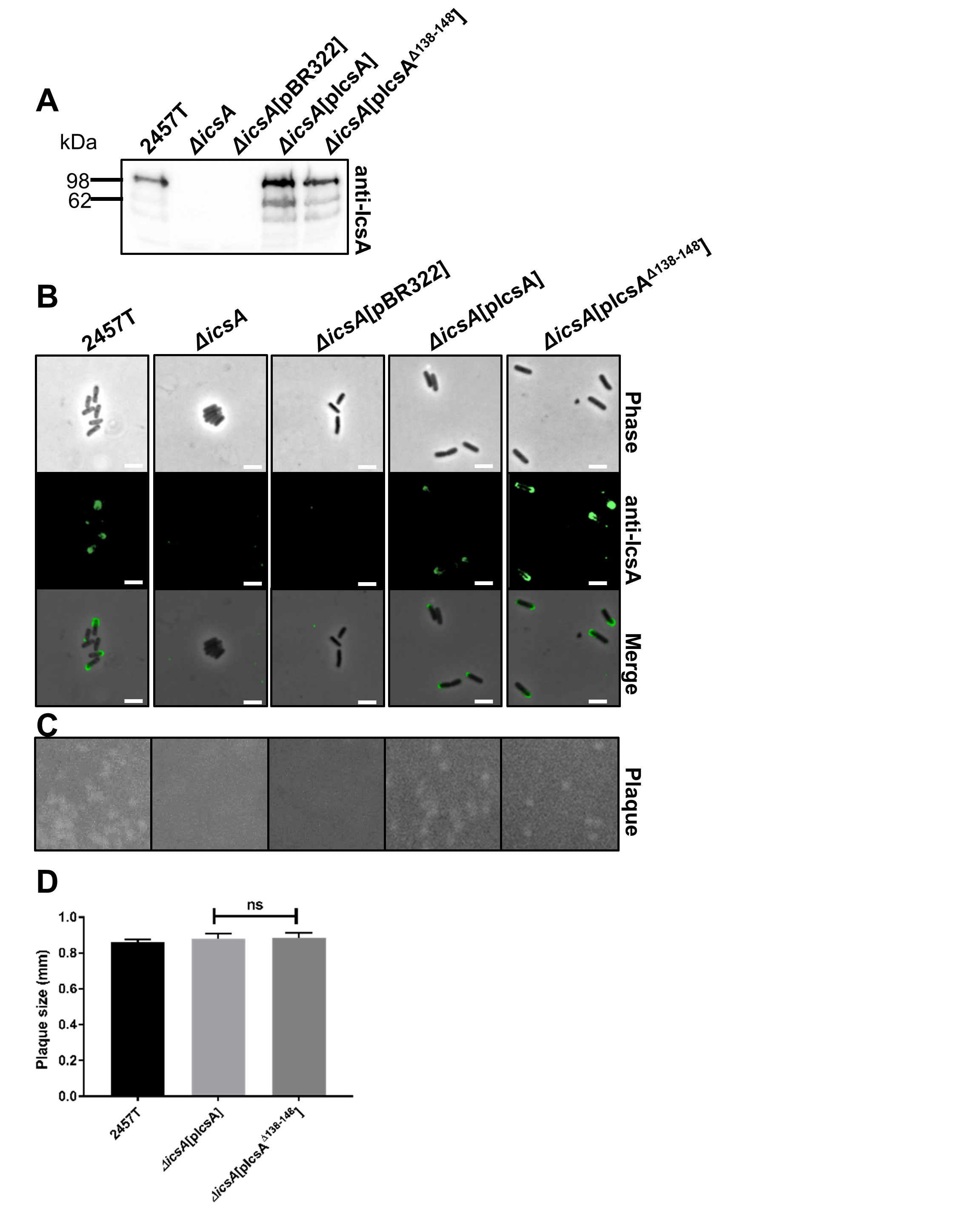

Supplement: S5 Fig — A. Western immunoblotting of S. flexneri 2457T, and ΔicsA expressing IcsA or IcsAΔ138–148. Shigella strains grown to an OD600 of 0.5 were collected and analysed via a 12% SDS-PAGE gel and Western immunoblotting with anti-IcsA. B. Immunofluorescent staining of IcsA with whole Shigella bacteria. Bacteria grown to an OD600 of 0.5 were collected and fixed with formaldehyde. IcsA was stained with rabbit anti-IcsA, and Alexa Fluor 488 conjugated donkey anti-rabbit antibodies. Images were acquired using an Olympus epifluorescence microscope [24]. Scale bar represents 2 μm. C. Plaque formation assay with IcsA mutants and their complemented strains. Shigella grown to an OD600 of 0.5 were collected to infect HeLa cell monolayers. After 1.5 h infection, the extracellular bacteria was killed by adding DMEM supplemented with 0.5% (w/v) agar and 40 μg/ml gentamycin. After 24 h post-infection, a second layer of DMEM medium containing 0.5% (w/v) agar and 0.1% (w/v) Neutral Red was added and images were taken after 72 h post-infection. D. Plaque size measurements for plaques formed in C. Data were acquired at least from 20 plaques for each strain and significance was calculated using a student t test, and p values are as follow: ns, non-significant. Note that ΔicsA and ΔicsA [pBR322] did not form plaques. (TIF) [file pone.0227425.s005.tif]

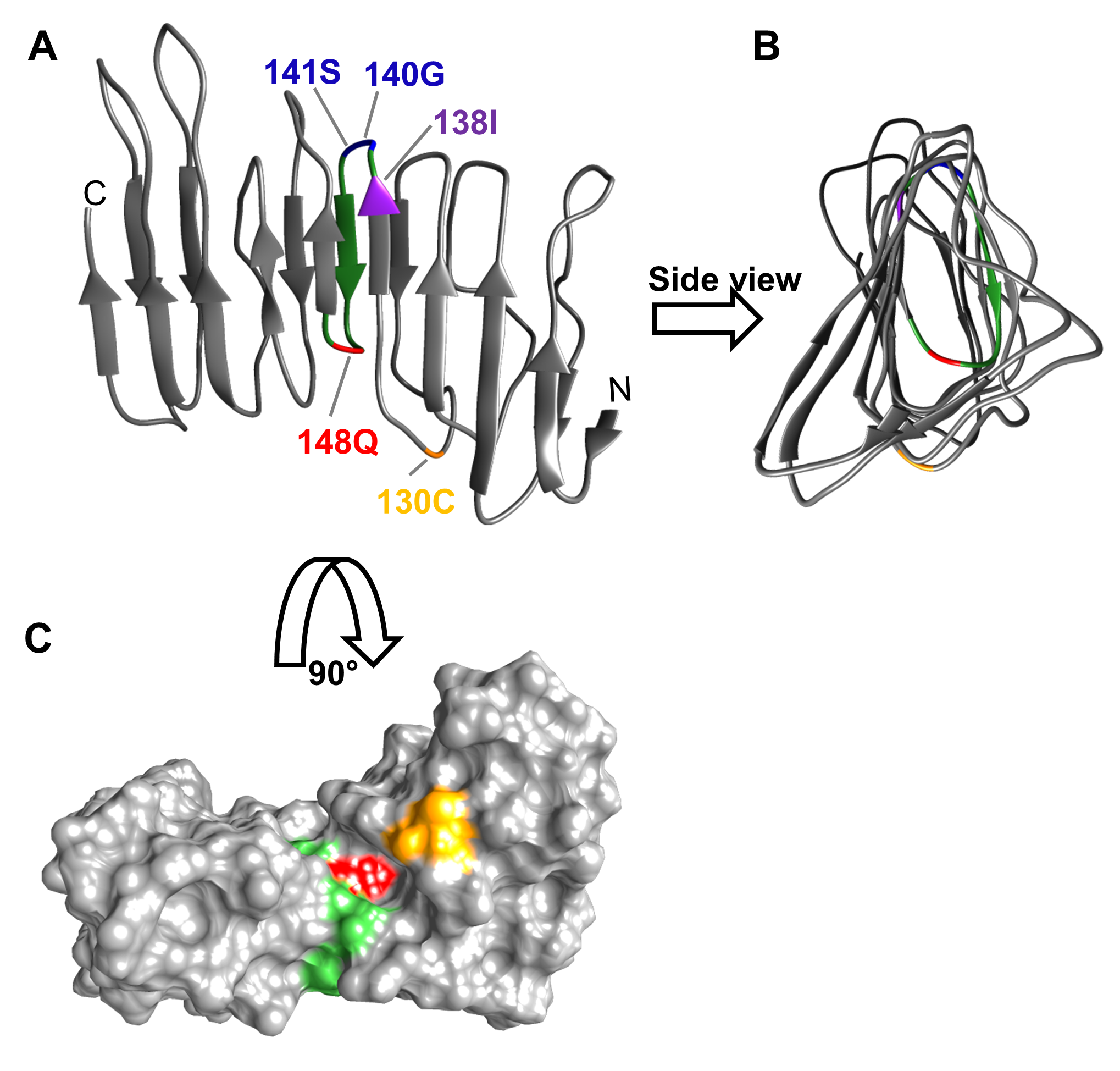

Supplement: S6 Fig — A. Predicted structure of IcsA passenger 55–241 shown in ribbon. B. Side view of the ribbon structure of IcsA55-241. C. Surface of the predicted IcsA55-241 structure. The structure of the IcsA passenger (55–241) was acquired from Itasser and annotated using Chimera. The amino group adjacent to the insertion sites (i138, i140 and i148) are marked on the structure. The IcsA adherent region is shown in green. (TIF) [file pone.0227425.s006.tif]
