# Supplementary material for: The virulence domain of Shigella IcsA contains a subregion with specific host cell adhesion function
Source: PLoS One. 2020 Jan 7;15(1):e0227425. doi: 10.1371/journal.pone.0227425 (PMC6946128; doi:10.1371/journal.pone.0227425)
Supplement: S1 Raw Images — (PDF) [file pone.0227425.s007.pdf]

Fig 2A

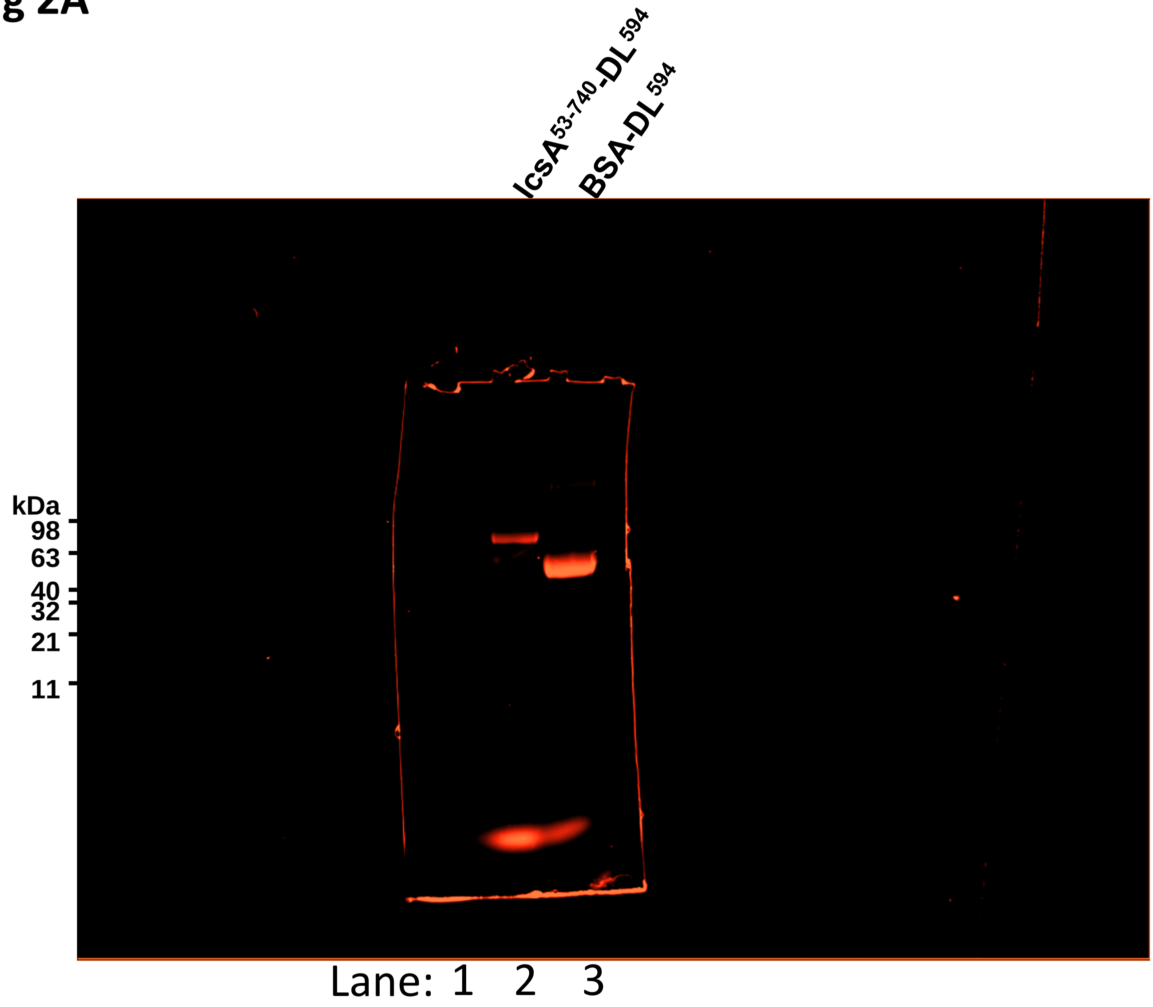

Original image of Fig 2A
Lane 2 and 3 were used in Fig 2A
Image was taken by an Universal Hood III (BioRad) under the channel of Dylight 650.
The Dylight594 labelled protein samples are as indicated

Fig 3. A

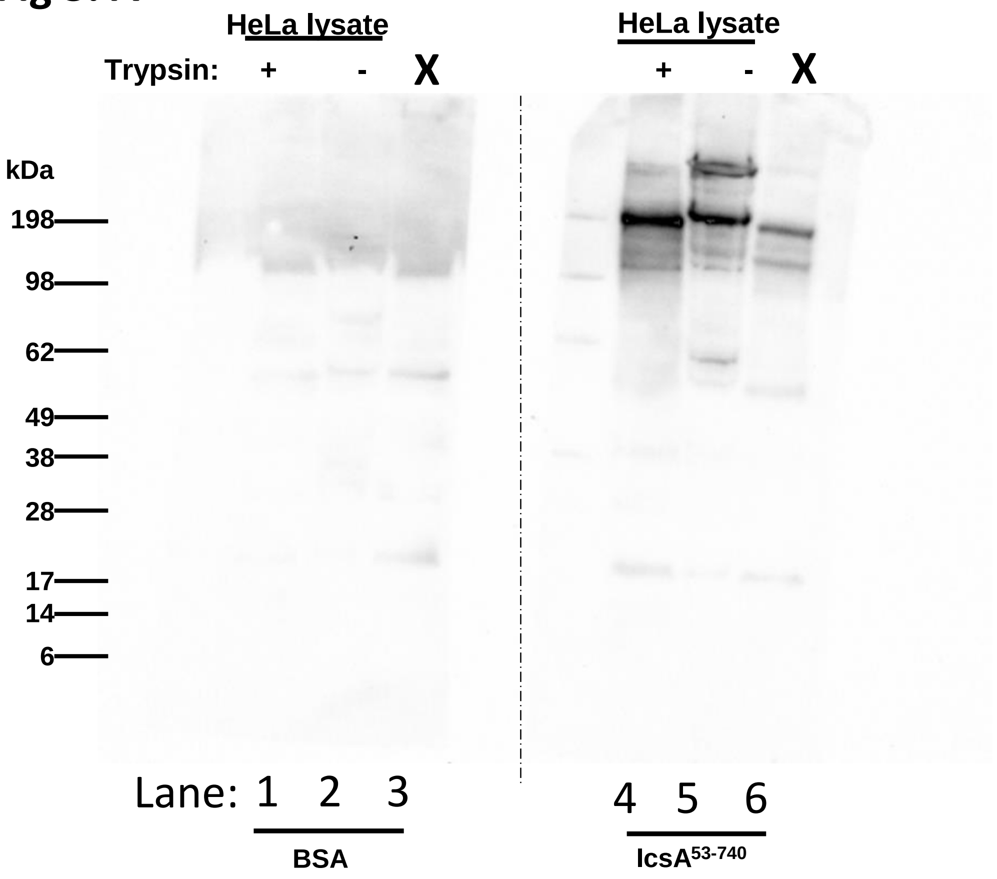

Original image of Fig 3.A
Two separate membranes incubated with either IcsA or BSA were imaged together to control the exposure, as indicated by the dashed line.
Lane 1-2 and 4-5 were used in Fig 3.A
Image was taken by an Universal Hood III (BioRad) under the channel of Chemi.
Far Wstern blot samples were as indicated
Unrelated experiment samples were marked by “X”.

Fig 3. B

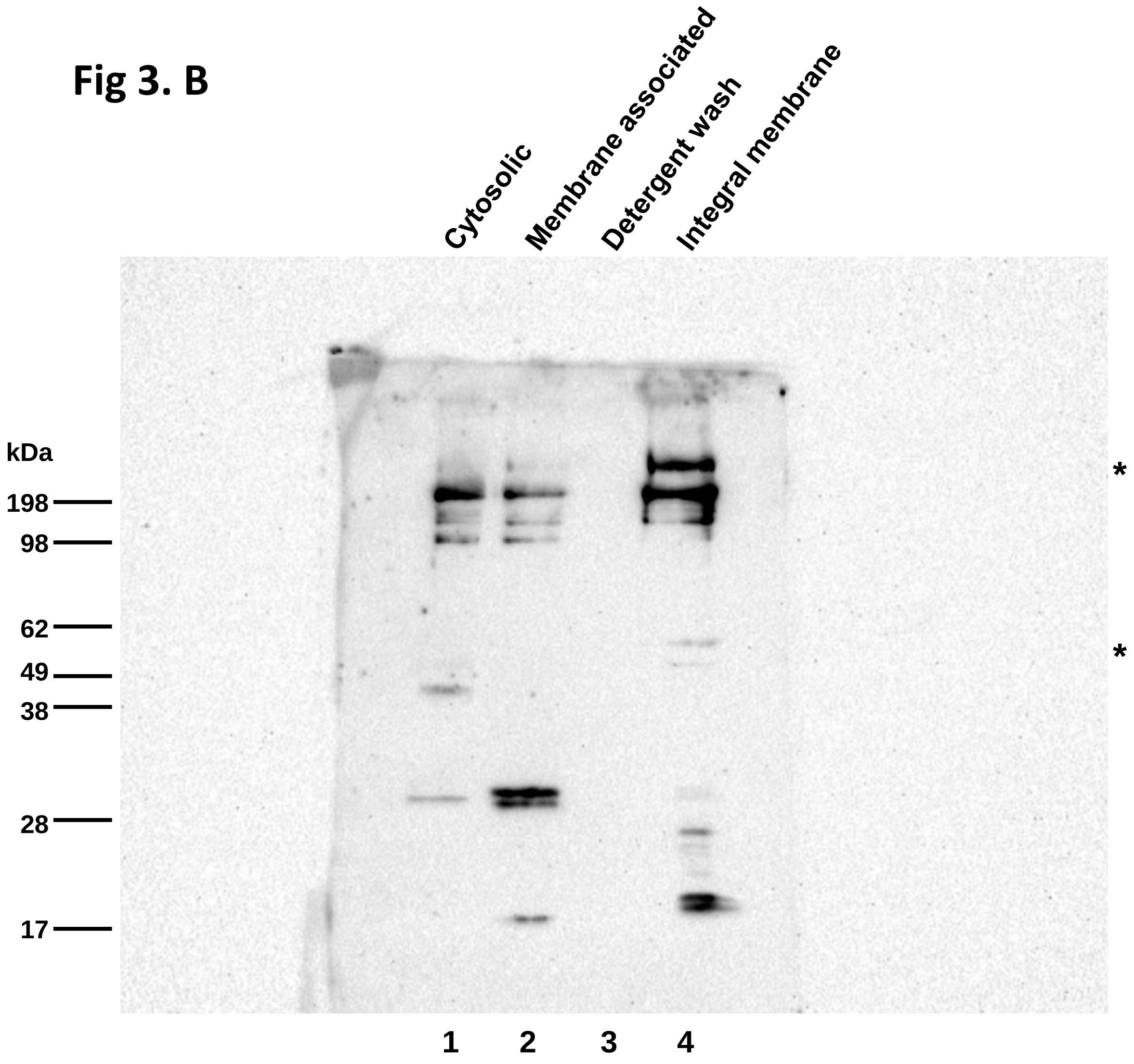

Original image of Fig 3B
The entire blot was used in Fig 3B
Image was taken by an Universal Hood III (BioRad) under the channel of Chemi.

Fig 5. B

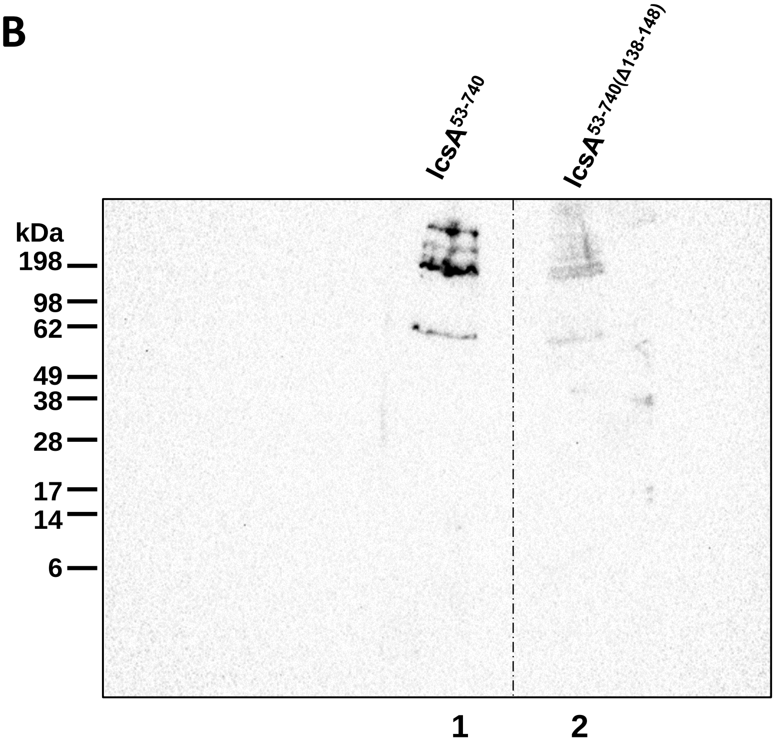

Original image of Fig 5B

Two separate membranes incubated with either IcsA$^{53\text{-}740}$ or IcsA$^{53\text{-}740(\Delta 138\text{-}148)}$ were imaged together to control the exposure, as indicated by the dashed line.

The entire blot was used in Fig 5B

Image was taken by an Universal Hood III (BioRad) under the channel of Chemi.

Far Western blot samples were as indicated

Fig 6A

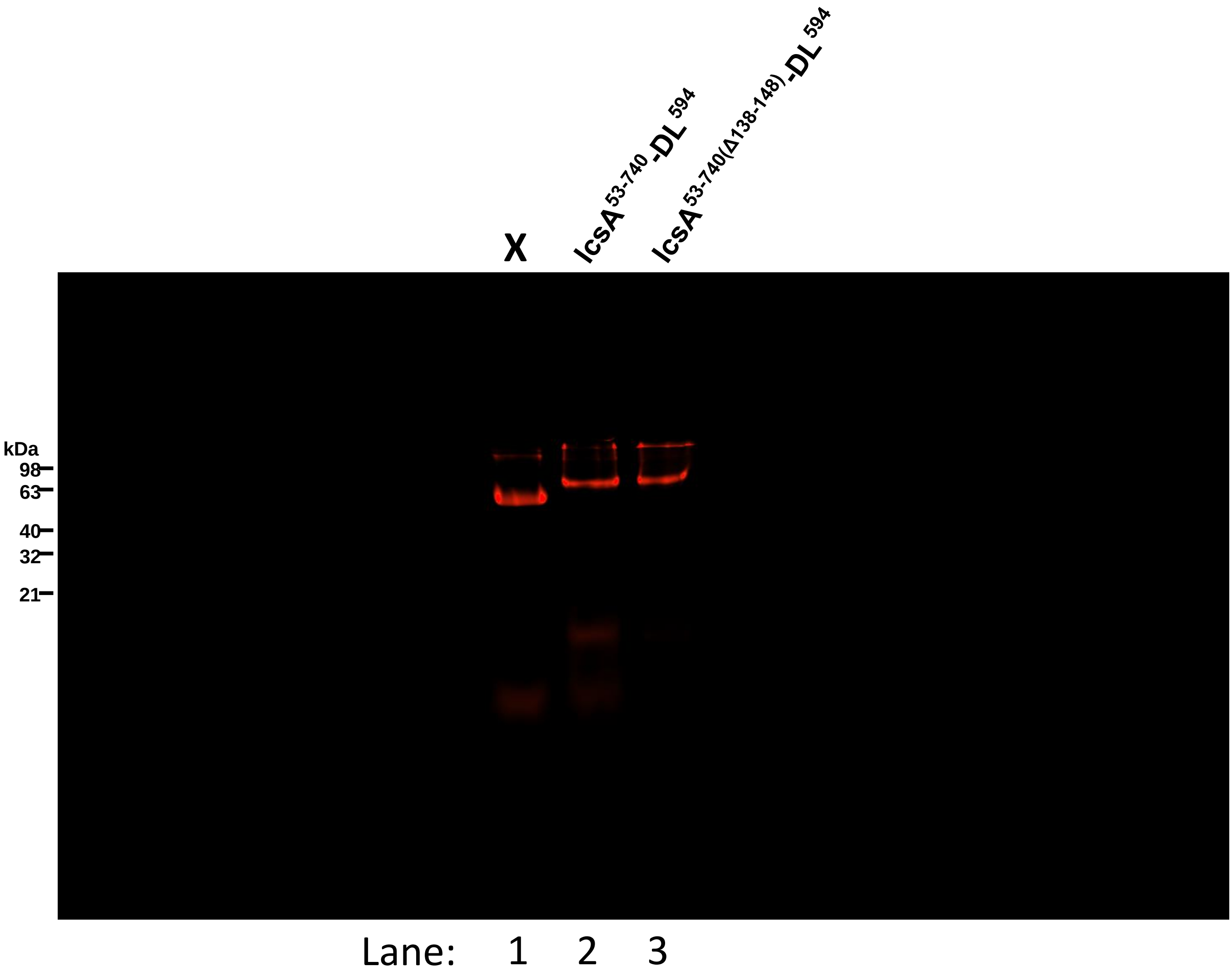

Original image of Fig 6A
Lane 2 and 3 were used in Fig 6A
Image was taken by an Universal Hood III (BioRad) under the channel of Dylight 650.
The Dylight594 labelled protein samples are as indicated
Unrelated experiment samples were marked by “X”.

S1 Fig. B

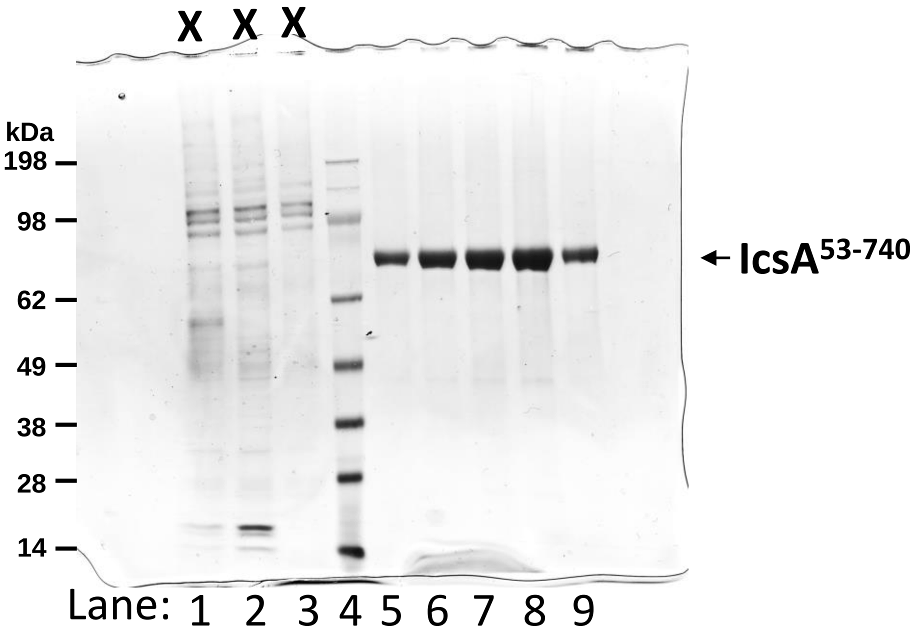

Original image of S1 Fig. B

Lane 4-9 which includes marker and purified IcsA protein were used in S1 Fig. B

The area of the gel was scanned by a HP scanner and saved as a tif image.

Unrelated experiment samples were marked by “X”.

S1 Fig. C

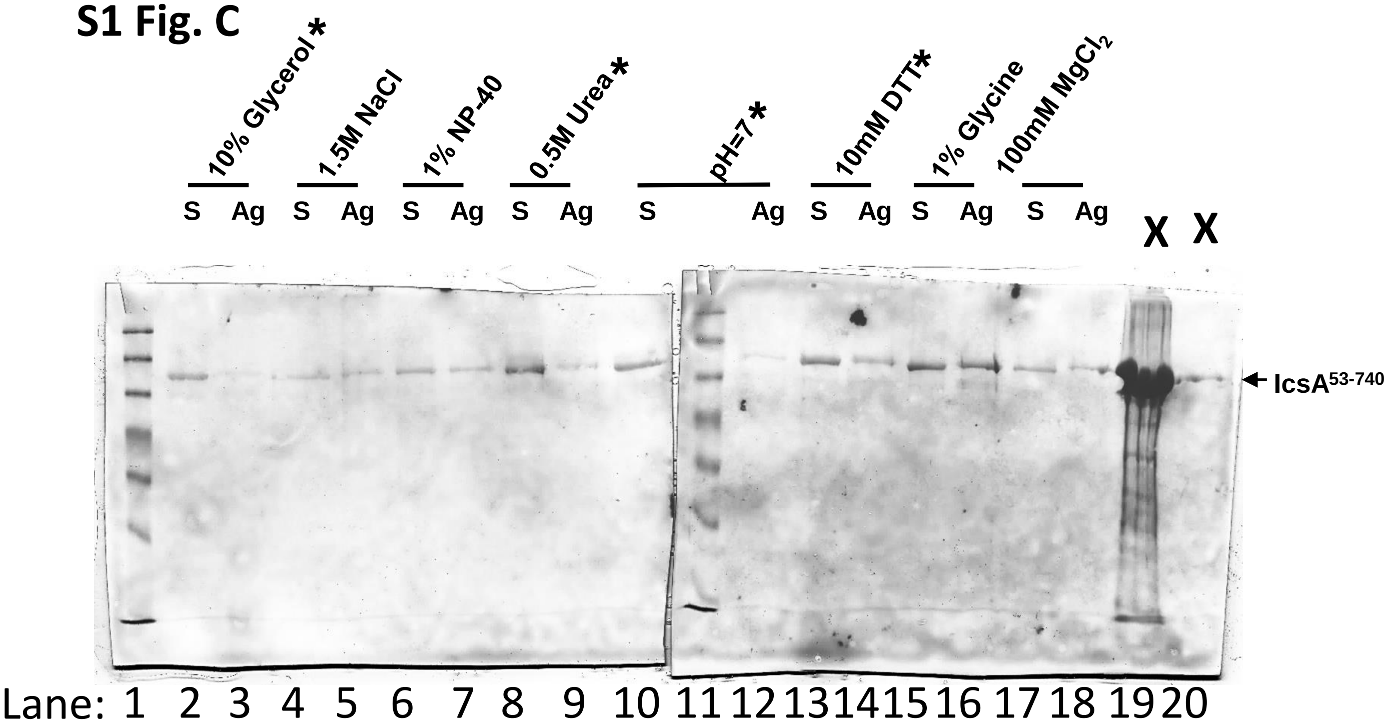

Original image of S1 Fig. C

Lane 1-10 and 12-18 which includes marker and purified IcsA protein in different conditions were used in S1 Fig. C

The area of the two membranes was scanned by a HP scanner and saved as a tif image.

Samples are as indicated.

Unrelated experiment samples were marked by “X”.

S1 Fig. D

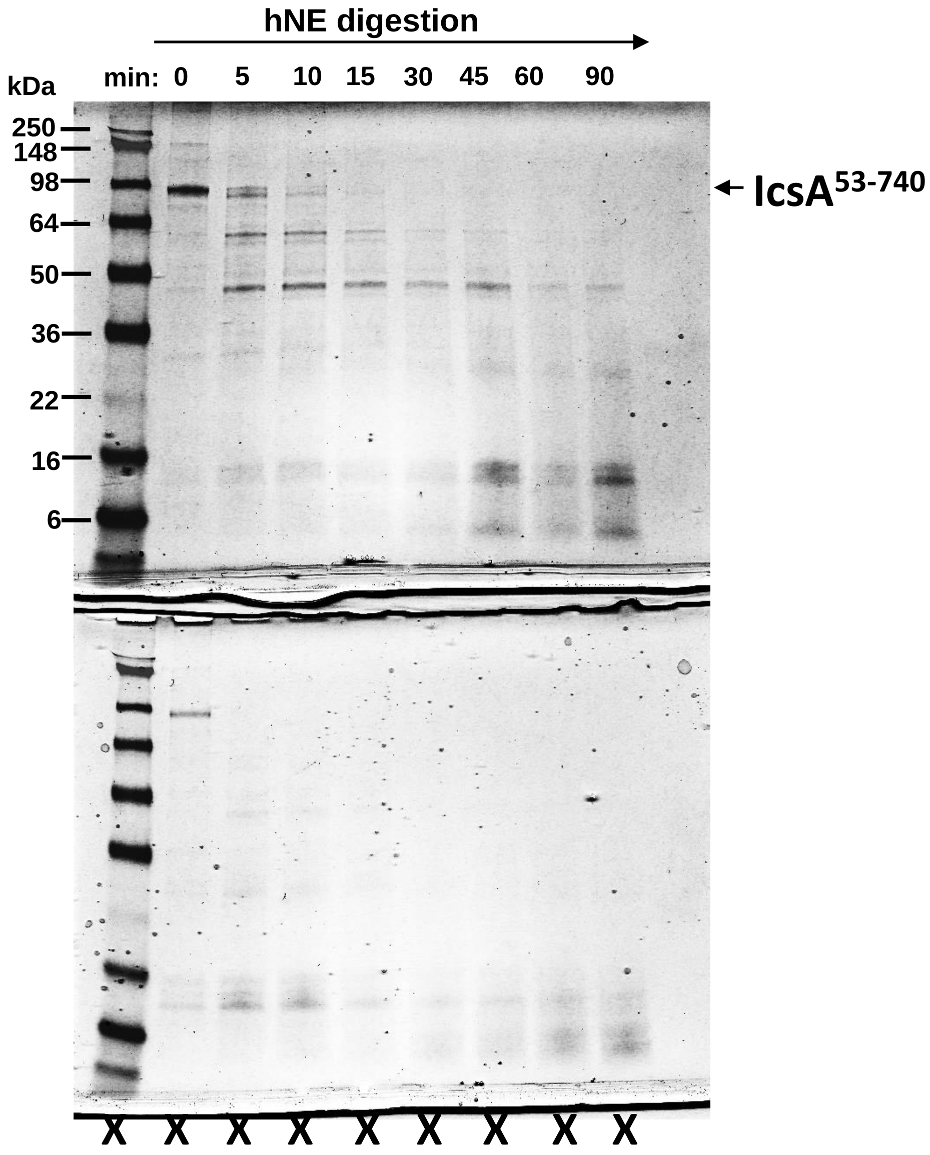

Original image of S1 Fig. D

The upper gel was used in S1 Fig. D

The area of the two gels was scanned by a HP scanner and saved as a tif image.

The digested IcsA samples are as indicated

Unrelated experiment samples were marked by “X”.

S1 Fig. E

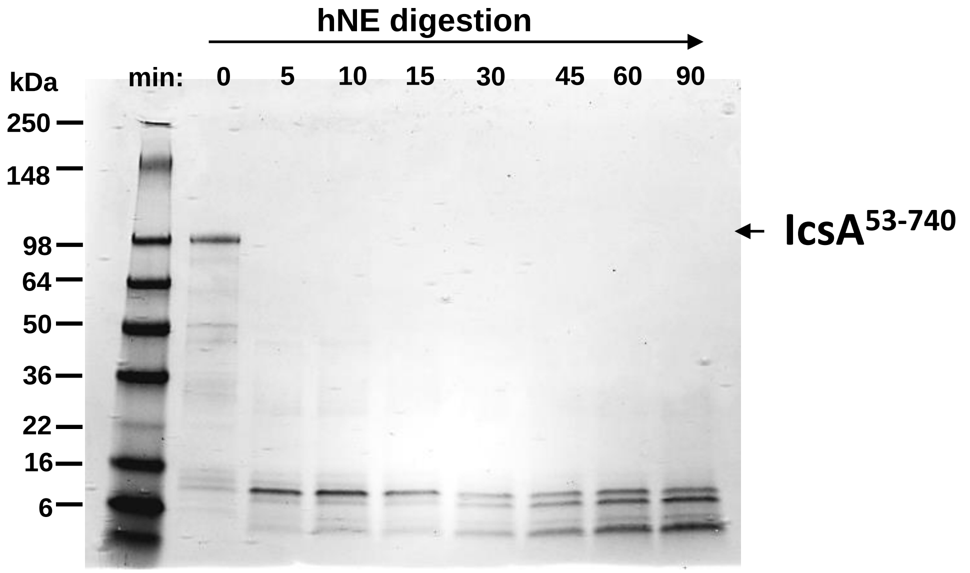

Original image of S1 Fig. E
The entire gel was used in S1 Fig. E
The area of the gel was scanned by a HP scanner and saved as a tif image.
The digested IcsA samples are as indicated

S2 Fig

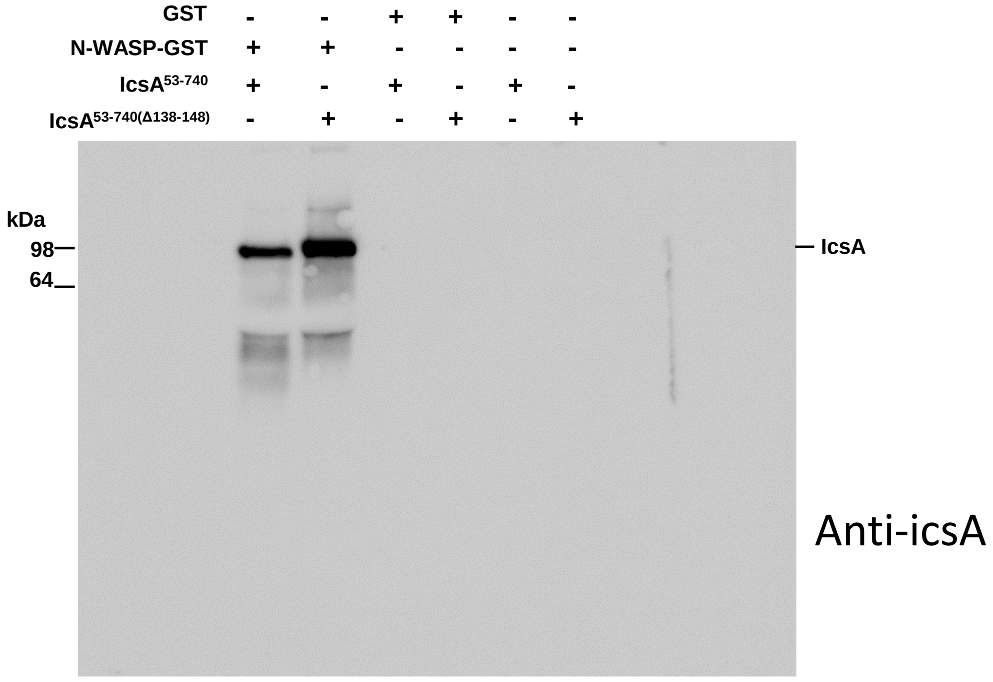

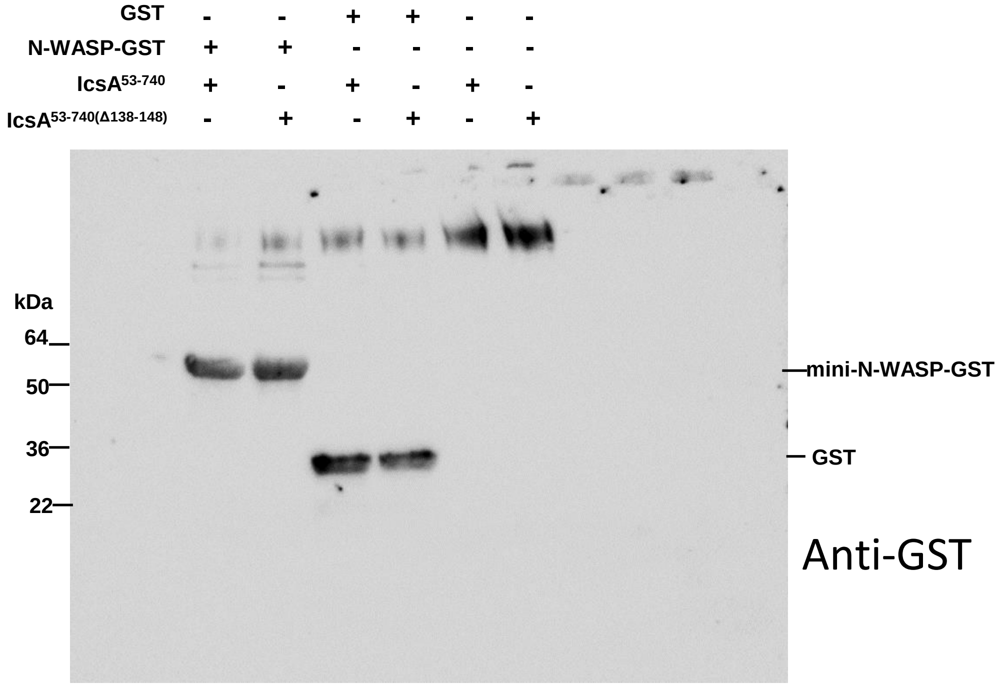

Original image of S2 Fig

Two blots were imaged separately and were used in S2 Fig

Image was taken by an Universal Hood III (BioRad) under the channel of Chemi.

Samples were as indicated

S5 Fig. A

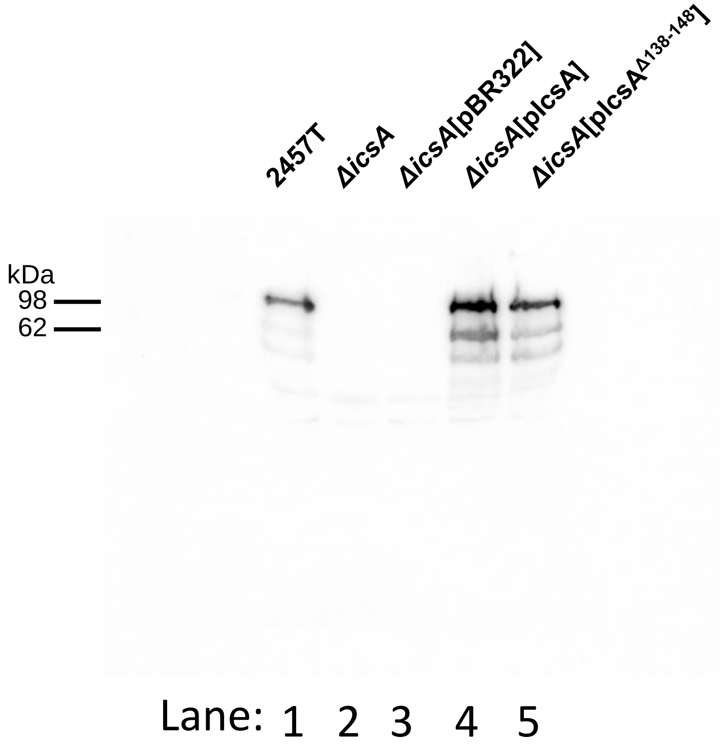

Original image of S5 Fig A
Lane 1-5 were used in S5 FigA
Image was taken by an Universal Hood III (BioRad) under the channel of Chemi.
Samples were as indicated
