# Supplementary material for: The virulence domain of Shigella IcsA contains a subregion with specific host cell adhesion function
Source: PLoS One. 2020 Jan 7;15(1):e0227425. doi: 10.1371/journal.pone.0227425 (PMC6946128; doi:10.1371/journal.pone.0227425)
Supplement: S1 Table — (PDF) [file pone.0227425.s008.pdf]

S1 Table. Strains and Plasmids

Strain or plasmid	Characteristics	Source/reference
Strains		
2457T	Wild type *S. flexneri* 2a	(1)
RMA2041	2457T*ΔicsA::Tc*^{R}	(2)
RMA2090	2457T*ΔicsA::Tc*^{R}[pIcsA]	(3)
JQRM9	2457T*ΔipaD*	This study
JQRM10	2457T*ΔipaB*	This study
JQRM11	2457T*ΔipaDΔicsA::Tc*^{R}	This study
JQRM12	2457T*ΔipaBΔicsA::Tc*^{R}	This study
MDRM190	TOP10[pMDBADIcsA$^{53\text{-}740}$]	This study
JQRM116	TOP10[pMDBAD-*icsA*$^{\Delta 138\text{-}148}$]	This study
Shigella IcsA 5aa insertion collection		
JQRM16	2457T*ΔicsAΔipaD::frt*[pIcsA]	This study
JQRM17	2457T*ΔicsAΔipaD::frt*[pBR322]	This study
JQRM22	2457T*ΔicsAΔipaD::frt*[pIcsA^{i56}]	This study
JQEM23	2457T*ΔicsAΔipaD::frt*[pIcsA^{i81}]	This study
JQRM24	2457T*ΔicsAΔipaD::frt*[pIcsA^{i87}]	This study
JQRM25	2457T*ΔicsAΔipaD::frt*[pIcsA^{i120}]	This study
JQRM26	2457T*ΔicsAΔipaD::frt*[pIcsA^{i122}]	This study
JQRM27	2457T*ΔicsAΔipaD::frt*[pIcsA^{i128}]	This study
JQRM28	2457T*ΔicsAΔipaD::frt*[pIcsA^{i132}]	This study
JQRM29	2457T*ΔicsAΔipaD::frt*[pIcsA^{i137}]	This study
JQRM30	2457T*ΔicsAΔipaD::frt*[pIcsA^{i138}]	This study
JQRM31	2457T*ΔicsAΔipaD::frt*[pIcsA^{i140}]	This study
JQRM32	2457T*ΔicsAΔipaD::frt*[pIcsA^{i148}]	This study
JQRM33	2457T*ΔicsAΔipaD::frt*[pIcsA^{i185}]	This study
JQRM34	2457T*ΔicsAΔipaD::frt*[pIcsA^{i193}]	This study
JQRM35	2457T*ΔicsAΔipaD::frt*[pIcsA^{i219}]	This study
JQRM36	2457T*ΔicsAΔipaD::frt*[pIcsA^{i226}]	This study
JQRM37	2457T*ΔicsAΔipaD::frt*[pIcsA^{i228}]	This study
JQRM38	2457T*ΔicsAΔipaD::frt*[pIcsA^{i230}]	This study
JQRM39	2457T*ΔicsAΔipaD::frt*[pIcsA^{i244}]	This study
JQRM40	2457T*ΔicsAΔipaD::frt*[pIcsA^{i248}]	This study
JQRM41	2457T*ΔicsAΔipaD::frt*[pIcsA^{i268}]	This study
JQRM42	2457T*ΔicsAΔipaD::frt*[pIcsA^{i271}]	This study
JQRM43	2457T*ΔicsAΔipaD::frt*[pIcsA^{i288}]	This study
JQRM44	2457T*ΔicsAΔipaD::frt*[pIcsA^{i292}]	This study
JQRM45	2457T*ΔicsAΔipaD::frt*[pIcsA^{i297}]	This study
JQRM46	2457T*ΔicsAΔipaD::frt*[pIcsA^{i312}]	This study
JQRM47	2457T*ΔicsAΔipaD::frt*[pIcsA^{i314}]	This study
JQRM48	2457T*ΔicsAΔipaD::frt*[pIcsA^{i322}]	This study
JQRM49	2457T*ΔicsAΔipaD::frt*[pIcsA^{i324}]	This study
JQRM50	2457T*ΔicsAΔipaD::frt*[pIcsA^{i326}]	This study
JQRM51	2457T*ΔicsAΔipaD::frt*[pIcsA^{i330a}]	This study
JQRM52	2457T*ΔicsAΔipaD::frt*[pIcsA^{i330b}]	This study
JQRM53	2457T*ΔicsAΔipaD::frt*[pIcsA^{i342}]	This study
JQRM54	2457T*ΔicsAΔipaD::frt*[pIcsA^{i346}]	This study
JQRM55	2457T*ΔicsAΔipaD::frt*[pIcsA^{i369}]	This study
JQRM56	2457T*ΔicsAΔipaD::frt*[pIcsA^{i381}]	This study
JQRM57	2457T*ΔicsAΔipaD::frt*[pIcsA^{i386}]	This study
JQRM58	2457T*ΔicsAΔipaD::frt*[pIcsA^{i456}]	This study
JQRM59	2457T*ΔicsAΔipaD::frt*[pIcsA^{i502}]	This study
JQRM60	2457T*ΔicsAΔipaD::frt*[pIcsA^{i532}]	This study

JQRM61	2457T*ΔicsAΔipaD::frt*[pIcsA^{i563}]	This study
JQRM62	2457T*ΔicsAΔipaD::frt*[pIcsA^{i595}]	This study
JQRM63	2457T*ΔicsAΔipaD::frt*[pIcsA^{i598}]	This study
JQRM64	2457T*ΔicsAΔipaD::frt*[pIcsA^{i633}]	This study
JQRM65	2457T*ΔicsAΔipaD::frt*[pIcsA^{i643}]	This study
JQRM66	2457T*ΔicsAΔipaD::frt*[pIcsA^{i677}]	This study
JQRM67	2457T*ΔicsAΔipaD::frt*[pIcsA^{i716}]	This study
JQRM68	2457T*ΔicsAΔipaD::frt*[pIcsA^{i748}]	This study
IcsA point mutants		
JQRM85	2457T*ΔicsAΔipaD::frt*[pIcsA$^{I138R/T139L}$]	This study
JQRM86	2457T*ΔicsAΔipaD::frt*[pIcsA$^{I138P/T139T}$]	This study
JQRM94	2457T*ΔicsAΔipaD::frt*[pIcsA$^{I138R/T139T}$]	This study
JQRM96	2457T*ΔicsAΔipaD::frt*[pIcsA$^{G140H/S141P}$]	This study
JQRM98	2457T*ΔicsAΔipaD::frt*[pIcsA$^{G140L/S141G}$]	This study
JQRM101	2457T*ΔicsAΔipaD::frt*[pIcsA$^{G140A/S140N}$]	This study
JQRM102	2457T*ΔicsAΔipaD::frt*[pIcsA$^{G140V/S140V}$]	This study
JQRM106	2457T*ΔicsAΔipaD::frt*[pIcsA$^{Q148D/G149S}$]	This study
JQRM107	2457T*ΔicsAΔipaD::frt*[pIcsA$^{Q148K/G149S}$]	This study
JQRM109	2457T*ΔicsAΔipaD::frt*[pIcsA$^{Q148F/G149Q}$]	This study
JQRM111	2457T*ΔicsAΔipaD::frt*[pIcsA$^{Q148C/G149N}$]	This study
Alanine scanning		
JQRM141	2457T*ΔicsAΔipaD::frt*[pIcsA^{138A}]	This study
JQRM142	2457T*ΔicsAΔipaD::frt*[pIcsA^{139A}]	This study
JQRM143	2457T*ΔicsAΔipaD::frt*[pIcsA^{140A}]	This study
JQRM144	2457T*ΔicsAΔipaD::frt*[pIcsA^{141A}]	This study
JQRM145	2457T*ΔicsAΔipaD::frt*[pIcsA^{142A}]	This study
JQRM146	2457T*ΔicsAΔipaD::frt*[pIcsA^{143A}]	This study
JQRM147	2457T*ΔicsAΔipaD::frt*[pIcsA^{144A}]	This study
JQRM148	2457T*ΔicsAΔipaD::frt*[pIcsA^{145A}]	This study
JQRM149	2457T*ΔicsAΔipaD::frt*[pIcsA^{146A}]	This study
JQRM150	2457T*ΔicsAΔipaD::frt*[pIcsA^{147A}]	This study
JQRM151	2457T*ΔicsAΔipaD::frt*[pIcsA^{148A}]	This study
JQRM152	2457T*ΔicsAΔipaD::frt*[pIcsA^{149A}]	This study
Plasmids		
pMDBADIcsA$^{53\text{-}740}$	IcsA$^{53\text{-}740}$ expression construct	This study
pIcsA	pBR322 derivatives with CDS of IcsA	(3)
pJQ9	pIcsA$^{\Delta 138\text{-}148}$ IcsA mutant	This study
pJQ11	pMDBAD::*icsA*$^{53\text{-}740/\Delta 138\text{-}148}$	This study
pKD46	Lambda red plasmid, Ap^R, 30 °C	(4)
pKD4	Vector containing FRT-flanked *kan*^{R} gene	(4)
pCP20	FLP recombinase, Ap^R, Cm^R, 30 °C	(4)

References

1. Van Den Bosch L, Manning PA, Morona R. 1997. Regulation of O-antigen chain length is required for *Shigella flexneri* virulence. Mol Microbiol 23:765-775.
2. Morona R, Van Den Bosch L. 2003. Multicopy icsA is able to suppress the virulence defect caused by *wzz*(SF) mutation in the *Shigella flexneri*. Fems Microbiol Lett 221:213-219.
3. Van den Bosch L, Morona R. 2003. The actin-based motility defect of a *Shigella flexneri rmlD* rough LPS mutant is not due to loss of IcsA polarity. Microb Pathog 35:11-8.
4. Datsenko KA, Wanner BL. 2000. One-step inactivation of chromosomal genes in *Escherichia coli* K-12 using PCR products. Proc Natl Acad Sci U S A 97:6640-5.
