# Supplementary material for: The virulence domain of Shigella IcsA contains a subregion with specific host cell adhesion function
Source: PLoS One. 2020 Jan 7;15(1):e0227425. doi: 10.1371/journal.pone.0227425 (PMC6946128; doi:10.1371/journal.pone.0227425)
Supplement: S2 Table — (PDF) [file pone.0227425.s009.pdf]

TABLE S2 Oligonucleotides

Oligos	Sequence[#]	Description
JQ1	TATATCCAAGAGCCATAATAATATATGGCTCTTCCTGTAAGGAAATAACCGTGTAGGCTGGAGCTGCTTC	*ΔipaD* Fwd
JQ2	GCCTTATATAAGAATGTTGGCGCTTGAGTATTATTTACATTATGCATGGCGCACCGCCATGGTCCATATGAATATCCTCC	*ΔipaD* Rev
JQ3	AAAGCACAATCATACTTGGACGCAATTCAGGATATCAAGGAGTAATTATTGTGTAGGCTGGAGCTGCTTC	*ΔipaB* Fwd
JQ4	TATAAAATCTGGGTTGGTTTTGTGTTTTGAATTTCCATAACATTCTCCTTATTTGTAGCCATGGTCCATATGAATATCCTCC	*ΔipaB* Rev
MD80	TTTTTTCTCGAGACTCCTCTTTCGGGTACTCAAG	XhoI $IcsA^{53}$ Fwd
MD81	TTTTTTGGTACCTTATCCATCTGACTAGTTAGATACCAC	KpnI $IcsA^{740}$ Rev
MD85	GGTATGGCTAGCATGACTGGTGG	His_{12} Fwd
MD86	ATGATGATGATGATGATGATGATGATGATGATGATGAGAACCCCCC	His_{12} Rev
JQ27	AATAGTTGTGGCGGTAATGGTGGTGACTCTNNNNNNGGATCTGACTTGTCTATAATCAATCAAGG	Site-directed mutagenesis primer targeting $IcsA_{138-139}$
JQ28	TGTGGCGGTAATGGTGGTGACTCTATTACCNNNNNNGACTTGTCTATAATCAATCAAGGCATG	Site-directed mutagenesis primer targeting $IcsA_{140-141}$
148NNS	ATTACCGGATCTGACTTGTCTATAATCAATNNSNNSATGATTCTTGGTGGTAGCGGCGGTAGCG	Site-directed mutagenesis primer targeting $IcsA_{148-149}$
JQ43	AGAGTCACCACCATTACCG	Universal primer for alanine scanning aa 138-149
JQ44	GCTACCGGATCTGACTTGTCTATAATC	Mutagenesis primer, I138A
JQ45	ATTGCTGGATCTGACTTGTCTATAATCAATCAAG	Mutagenesis primer, T139A
JQ46	ATTACCGCTTCTGACTTGTCTATAATCAATCAAGG	Mutagenesis primer, G140A
JQ47	ATTACCGGAGCTGACTTGTCTATAATCAATCAAGGCATG	Mutagenesis primer, S141A
JQ48	ATTACCGGATCTGCTTTGTCTATAATCAATCAAGGCATGATTC	Mutagenesis primer, D142A
JQ49	ATTACCGGATCTGACGCTTCTATAATCAATCAAGGCATGATTCTTG	Mutagenesis primer, L143A
JQ50	ATTACCGGATCTGACTTGGCTATAATCAATCAAGGCATGATTCTTGG	Mutagenesis primer, S144A
JQ51	ATTACCGGATCTGACTTGTCTGCTATCAATCAAGGCATGATTCTTGG	Mutagenesis primer, I145A
JQ52	ATTACCGGATCTGACTTGTCTATAGCTAATCAAGGCATGATTCTTGGTG	Mutagenesis primer, I146A
JQ53	ATTACCGGATCTGACTTGTCTATAATCGCTCAAGGCATGATTCTTGGTGG	Mutagenesis primer, N147A
JQ54	ATTACCGGATCTGACTTGTCTATAATCAATGCTGGCATGATTCTTGGTGGTAG	Mutagenesis primer, Q148A

JQ55	ATTACCGGATCTGACTTGTCTATAATCAATCAAGCTATGATTCTTGGTGGTAGCGG	Mutagenesis primer, G149A

underlined nucleotides are degenerated nucleotides.
